# Differential associations of the timing and amount of social media use with sleep quality and academic performance among undergraduate medical and dental students in Pakistan: a cross-sectional study

**DOI:** 10.3389/fpubh.2026.1968305

**Published:** 2026-09-28

**Authors:** Kanza Atif, Abdal Ahmad, Ayat Ullah Khan, Aziz Khan, Ata Ullah, Muhammad Umair Amjad, Muhammad Tafheem Khan, Muhammad Kakakhel, Muhammad Qasim, Muhammad Talha Bin Shakoor, Abdul Latif, Sadia Qazi

**Affiliations:** 1 College of Medicine, Alfaisal University, Riyadh, Saudi Arabia; 2 Peshawar Medical College, Riphah International University, Peshawar, Khyber Pakhtunkhwa, Pakistan; 3 Saidu Teaching Hospital, Saidu Sharif, Swat, Khyber Pakhtunkhwa, Pakistan; 4 King Abdullah Teaching Hospital, Mansehra, Khyber Pakhtunkhwa, Pakistan; 5 Khyber Teaching Hospital, Peshawar, Khyber Pakhtunkhwa, Pakistan; 6 Department of Anatomy, College of Medicine, Alfaisal University, Riyadh, Saudi Arabia

**Keywords:** academic performance, dental students, medical students, nocturnal smartphone use, Pakistan, Pittsburgh Sleep Quality Index, sleep quality, social media

## Abstract

**Background:**

Poor sleep and reduced academic performance among medical and dental students have been linked to social media use; however, whether the amount, purpose, and timing of use are independently associated with these outcomes remains unclear.

**Methods:**

In this cross-sectional study, 364 students enrolled in MBBS or BDS programs at ten institutions in Khyber Pakhtunkhwa, Pakistan, were recruited through convenience sampling and completed a paper-based questionnaire between October 2023 and April 2024. Sleep quality was measured using the Pittsburgh Sleep Quality Index (PSQI), and academic performance was assessed using self-reported percentage marks from the most recent professional examination. The models were adjusted for background characteristics and other social media exposures.

**Results:**

Poor sleep quality (PSQI > 5) was present in 312 students (85.7%, 95% CI 81.7–88.9). Duration of pre-sleep social media use was the only exposure independently associated with the PSQI global score (B = 1.594, 95% CI 1.081 to 2.106, *p* < 0.001), while total daily use was not. Conversely, each one-category increase in total daily use was associated with examination scores that were 1.40 percentage points lower (B = −1.404, 95% CI −2.053 to −0.756, *p* < 0.001). The classification of platform-derived purpose was not associated with either outcome; however, a secondary measure of purpose, the proportion of use devoted to academic work, was associated with a higher examination percentage (B = 2.148, 95% CI 1.513 to 2.782, *p* < 0.001). Thus, conclusions about purpose depended on how it was measured. Nocturnal checking was not associated with either outcome. Fitting both outcomes in a single model supported outcome specificity, most clearly for pre-sleep use (difference in standardized β = −0.271, *p* = 0.006; total daily use, 0.235, *p* = 0.023).

**Conclusion:**

Sleep quality was primarily associated with when social media was used, while examination performance was related to how much social media was used. These exploratory associations from a convenience sample cannot establish causal direction, apply to the participating institutions rather than to Pakistani students generally, and require replication with prospective designs and objective measures in future studies.

## 1 Introduction

Insufficient and poor-quality sleep is a modifiable determinant of health, cognitive function, and safety in young adults. Among students, its prevalence is high enough to constitute a population health concern in its own right, with international meta-analyses reporting a high pooled prevalence of poor sleep quality among medical students (1), most recently 59.1% across the Middle East and North Africa (2). In the health professions, the consequences extend beyond the individual because impaired attention, memory, and emotional regulation affect learning and, later, clinical performance. Social media use is one of the few candidate determinants of student sleep that is both highly prevalent and directly modifiable, and its role is complex. These platforms facilitate communication, academic discussion, and access to learning resources, as well as entertainment, news, passive scrolling, and peer interaction (3–6). Medical and dental students are a particularly relevant group because their training demands sustained attention and self-regulation under heavy examination pressure; however, evidence from low- and middle-income settings, where most of the world’s health workforce is trained, remains sparse.

When used for clear academic purposes, social media can support self-regulated learning and is now widely integrated into medical education (3, 4). It is frequently argued that associations with outcomes depend on how social media is used rather than on exposure alone; prolonged or academically intrusive use has been linked to academic performance (5), and concerns have been raised about addiction-like engagement (6, 7). Distraction is the principal proposed pathway, with notifications, boredom, and multitasking during study time fragmenting the sustained attention required for medical learning. Social media addiction has been associated with academic procrastination among medical students in Peshawar (8).

Sleep quality is a parallel concern, and the two outcomes have been studied together: both smartphone addiction and sleep quality have been associated with academic performance (9), sleep has been identified as an important determinant of achievement among medical students (10), and a study at a Pakistani medical university linked poor sleep quality to lower academic performance (11). Nighttime use may matter more than total daily volume: poor sleep has been reported among students who use smartphones at bedtime (12) and among those with excessive Internet use (13). Additionally, social media addiction, fatigue, and fear of missing out (14), as well as nighttime and multi-platform use (15), have been associated with poorer sleep. Checking social media after waking during the night is a distinct behavior that may further fragment sleep, yet it is rarely measured separately.

Platforms also differ in their primary uses: WhatsApp mainly supports peer communication and academic coordination, while YouTube is used for lecture videos. In contrast, Instagram, TikTok, and Snapchat are more closely tied to entertainment and passive scrolling, Twitter/X to news, and LinkedIn to professional networking. Collapsing these into a single measure has been argued to obscure meaningful differences, as academic use may relate to performance differently from entertainment use, and planned daytime use may not relate to sleep in the same way that passive scrolling before bed does (3, 4, 15).

Three features of this evidence base shape the present study. First, the behaviors most plausibly linked to sleep appear to carry the signal: a systematic review confined to 16–25-year-olds found that most studies of bedtime or nighttime use reported associations with poor sleep (16), a survey separating evening from after-bedtime use found risk concentrated in the latter (17), evening and bedtime smartphone use have been reported to be associated with sleep quality independently of total usage time (18), and in an international survey of medical students, screen use after nocturnal awakenings remained independently associated with poor sleep while smartphone addiction symptoms did not (19). Second, the evidence on aggregate volume is mixed and outcome-specific: total daily use was not associated with objectively measured sleep in a 14-day accelerometry study (20) and was unrelated to sleep problems after adjustment in a four-country survey (21), whereas a systematic review of problematic social media use and academic performance excluded studies measuring exposure only as time spent or frequency of use, on the grounds that these do not capture problematic use as a construct (22), leaving open whether volume behaves differently for sleep than for academic achievement. Third, the mechanisms have been formalized as bright light, arousal, nighttime disruption, and sleep displacement, along with bidirectional pathways in which technology is used to fill time or regulate emotion at sleep onset (23).

We therefore make no claim that amount, purpose, timing, or nocturnal checking are novel exposures; each has been examined before, including in multivariable models (14, 15, 18, 19). However, it is unclear whether these dimensions are related to different outcomes. We are not aware of a study that tests all three against both outcomes within the same adjusted model, which is necessary to show that sleep and academic performance are associated with different aspects of the same behavior rather than a common underlying intensity of use. Evidence from Pakistani undergraduate medical and dental students is also limited, and dental students have rarely been studied. We examined whether the amount, purpose, and timing of social media use are independently associated with sleep quality and academic performance among undergraduate medical and dental students in Khyber Pakhtunkhwa, Pakistan, and specifically whether the two outcomes are associated with the same or different aspects of use.

## 2 Materials and methods

### 2.1 Study design and setting

A cross-sectional study was conducted between October 2023 and April 2024 at 10 undergraduate medical and dental institutions in Khyber Pakhtunkhwa province, Pakistan, and is reported in accordance with the STROBE statement for cross-sectional studies (24) (STROBE checklist in the Supplementary Material).

Seven medical colleges (Khyber Medical College, Khyber Girls Medical College, Peshawar Medical College, Rehman Medical College, Kabir Medical College, Pak International Medical College, and Jinnah Medical College) and three dental colleges (Khyber College of Dentistry, Peshawar Dental College, and Rehman College of Dentistry), both public and private, participated in the study. Data were collected at a single time point with no intervention or follow-up.

### 2.2 Participants and eligibility

The target population comprised students enrolled in Bachelor of Medicine, Bachelor of Surgery (MBBS), and Bachelor of Dental Surgery (BDS) programs at the participating institutions. Students in the second (pre-clinical), third, fourth, and final (clinical) years were eligible; first-year students were not included. Eligibility required current enrollment, willingness to participate, and completion of all core questionnaire sections.

### 2.3 Sample size

The study size was determined *a priori* for the prevalence objective and then checked against the requirements of the primary multivariable models. These address two different questions: the precision of the prevalence estimate and the smallest association the study could detect.

#### 2.3.1 Precision of the prevalence estimate

The required sample was calculated using Cochran’s formula (25), assuming 95% confidence (Z = 1.96), an expected proportion of 0.50 to maximize the required sample size, and a 5% absolute margin of error:


$$n_{0}=Z^{2}p(1-p)/e^{2}={\left(1.96\right)}^{2}(0.50)(0.50)/{\left(0.05\right)}^{2}=384.16$$

A finite population correction was applied using the total undergraduate enrollment across the ten participating institutions (*N* = 6,650).


$$n=n_{0}/[1+(n_{0}-1)/N]=384.16/[1+383.16/6,650]=363.2$$

Therefore, the *a priori* target was 364 completed questionnaires; without the finite population correction, the requirement was 385. Because sampling was non-probability (Section 2.4), the correction was nominal rather than design-based, and using the larger total enrollment denominator was conservative.

#### 2.3.2 Precision achieved

In the analytic sample of 364, the observed prevalence of poor sleep was 85.7%, which resulted in a 95% confidence interval half-width of 3.6 percentage points without the finite population correction and 3.5 with it, narrower than the planned 5 points. Section 3.3 reports the uncorrected Wilson score interval; full calculations are provided in Supplementary Note S2.

#### 2.3.3 Adequacy of the multivariable models

Each fully adjusted model contained 13 predictor degrees of freedom, yielding 28 participants per predictor and exceeding the conventional requirements for multiple linear regression of *N* ≥ 50 + 8 m = 154 for the overall model and *N* ≥ 104 + m = 117 for individual predictors (26). At α = 0.05 and 80% power, the minimum detectable effect was f^2^ = 0.051 for the omnibus F-test and f^2^ = 0.022 for an individual predictor; group difference calculations are in Supplementary Note S2. Program × exposure interaction tests were not separately powered and were exploratory.

#### 2.3.4 Adequacy of the logistic model

The binary sleep outcome was unfavorably distributed (312 poor sleepers, 52 good sleepers), yielding 4.0 events per variable, below the conventional minimum of 10 (27), although this threshold is not absolute (28). The continuous PSQI global score was therefore designated as the primary sleep outcome, with the > 5 dichotomy as secondary; odds ratios were interpreted as associations rather than precise effect sizes, and Firth’s penalized-likelihood refitting (29, 30) produced essentially unchanged estimates with no sign changes (Supplementary Table S7).

#### 2.3.5 Clustering

Participants were recruited from ten institutions, but the institution of enrollment was not recorded at the participant level; therefore, the intracluster correlation could not be estimated, and cluster-robust standard errors could not be calculated. The consequence is quantifiable in principle: with a mean of 36.4 participants per institution, the design effect is DEFF = 1 + (m̄ − 1)ρ (31), yielding 1.354 at an intracluster correlation of 0.01 and 1.708 at 0.02, with effective sample sizes of 269 and 213, respectively, and standard errors 16.4 and 30.7% larger than under the independence assumption used here. Therefore, every interval reported in this article is narrower, and every *p*-value is smaller than what a correctly clustered analysis would produce. Because the standard errors cannot be recovered, the standard error of every reported estimate was inflated by √DEFF under both assumed values of ρ, and the interval was recomputed (Section 3.11; Supplementary Table S12).

#### 2.3.6 Pilot

The questionnaire was pilot-tested on 36 students, approximately 10% of the intended analytic sample, meeting the accepted guidance for pilot work of this kind (32, 33). Pilot participants came from the same institutions, were not included in the analytic sample, and their responses were not used to estimate any parameters reported in this study.

### 2.4 Sampling, recruitment, and data collection

#### 2.4.1 Sampling design

Non-probability convenience sampling was used. No sampling frame was enumerated, and no randomization was applied at the institution, class, or individual level; therefore, the achieved sample reflects those who were accessible during the recruitment window, and the design was not intended to yield a province-representative sample. Site-level quotas of approximately 30 to 45 completed questionnaires were set in advance to obtain broadly comparable representation across the ten institutions rather than in proportion to cohort size. Recruitment continued at each site until the quota was met and stopped once returns exceeded the target of 364. Completeness screening was applied afterward, at data entry, so the size of the analytic sample was unknown during fieldwork.

#### 2.4.2 Field procedures

Data were collected by seven members of the research team, all medical students, who were briefed beforehand on a common recruitment script to ensure that the study was described identically at every site. The collectors attended each campus in person and approached students individually or in small groups between scheduled teaching sessions, explaining the purpose of the study, its voluntary nature, the anonymity of responses, and the right to decline or stop at any point without academic consequence. Students who agreed completed a paper questionnaire unaided in 12–15 min and returned it on the same day. The collector answered questions about item wording but did not assist with or observe individual responses to the questions. No incentive was offered, and no questionnaire was completed online, by proxy, or by interview.

#### 2.4.3 Anonymity and handling of the completed forms

No identifiers were recorded on the questionnaire, and no signed consent form was collected (Section 2.7); therefore, no document existed that could link a participant to a returned questionnaire. Completed forms were collected in random order and pooled before a sequential study identifier was assigned and stored in a locked cabinet at Peshawar Medical College, accessible only to the research team.

#### 2.4.4 Participant flow

Of the 391 students approached, 12 declined to participate, and 379 questionnaires were returned, resulting in a response rate of 96.9%. Completeness screening excluded 15 forms with one or more incomplete core sections, yielding 364 complete responses (96.0% of returns and 93.1% of those approached). A core section included any of the demographic, examination percentage, social media exposure, timing and nighttime use, or sleep quality sections. Because forms with any incomplete core section were excluded at entry, the analytic dataset contained no item-level missing values, and complete-case analysis was used throughout. The 15 excluded questionnaires could not be compared with those retained, as the anonymous design recorded no information on them, and recruitment logs were maintained for the study as a whole rather than by site; therefore, non-participation could not be examined for site-level clustering.

#### 2.4.5 Data entry and verification

Responses were transcribed once from paper forms into a single spreadsheet, which was screened in full for out-of-range values, internal inconsistencies between related items, and duplicate records. Every flagged entry was checked against the source questionnaire before locking the dataset. Independent duplicate entry was not performed, and no random subsample was re-entered (Section 4.9). PSQI component and global scores were derived by script from the raw item responses using the published algorithm (34) rather than being entered manually, and the derivation was verified against a manually scored random subsample.

### 2.5 Instrument

The questionnaire comprised eight sections: demographic and academic information, social media volume and platform use, purpose-specific use, study-time multitasking, timing and nighttime use, sleep quality, digital behavior in relation to future clinical roles, and self-regulation and perceived impact. The response options and coding for every variable analyzed here, along with item wording where it was recorded verbatim, are presented in Supplementary Table S13. The text below describes their structures.

#### 2.5.1 Development and pilot testing

No validated instrument covering platform-specific purposes, study-time multitasking, and nocturnal checking within a single tool was available; therefore, these sections were developed for this study. Items were drafted from constructs identified in the literature and reviewed for clarity, relevance, and non-redundancy by the research team and medical education experts, and face and content validity were assessed through expert review. The pilot described in Section 2.3 assessed item clarity, adequacy of response options, and completion time, and the item wording was refined accordingly.

Reliability was reported for each multi-item measure used in the analysis: the PSQI (α = 0.779, computed across the seven component scores rather than the raw items) and the 2.5.4 Problematic Use Composite (α = 0.807). The five behavioral exposures are single-indicator measures for which internal consistency coefficients do not apply.

#### 2.5.2 Demographic and academic variables

Age was recorded in completed years, and sex was categorized as male, female, or prefer not to say; all 364 participants selected male or female, so the variable was analyzed dichotomously, and gender identity was not assessed. The year of study was recorded in four categories (second year, pre-clinicals; third, fourth, and final years, clinical), program as MBBS or BDS, institution type as public or private, and living arrangement as hostel or on-campus versus off-campus or with family.

Participants reported the percentage mark obtained in their most recent professional examination as a free-text value on a 0–100 scale, with the instruction not to round to a band. This was the only academic item analyzed as an outcome measure. Perceived class rank, satisfaction with performance, and perceived interference of social media with study were reported descriptively.

#### 2.5.3 Social media exposures

All 364 participants reported using social media, so no participant followed the filter that would have directed a non-user past these sections. Five behavioral exposures were analyzed: four are described here, and the fifth is the purpose of use. Total daily use across all platforms was recorded in six ordered bands, from under 30 min to more than 5 h, and coded as 1–6. Study-time multitasking was recorded on a five-point frequency scale, and the duration of pre-sleep social media use was recorded in five ordered bands. In each case, the lowest category, never using social media while studying, and no use in the last hour before sleep, was selected by no participants; therefore, both analyzed variables ranged from 1 to 4. Nocturnal checking, the frequency of checking social media upon waking during the night, was recorded in four categories coded 1–4. A further measure, the proportion of total social media time devoted to academic or study-related purposes, was recorded in six ordered bands coded 1–6. It is reported in the correlation matrix as the academic time percentage and was examined in a secondary, mutually adjusted analysis. Platform use was assessed using a multiple-response item covering eight named platforms plus a free-text option, from which the number of platforms used was derived as a count from 0 to 8.

The purpose of use was derived from a per-platform primary purpose item by comparing the number of platforms nominated as academic with the number nominated as entertainment for each participant. Participants with more academic than entertainment nominations were classified as academic, those with more entertainment than academic nominations as entertainment-dominated, and those with equal non-zero counts as mixed nominations. No participant was left unclassified, and this rule reproduced the purpose variable in the analysis dataset for all 364 participants in the study. The derivation rule, handling of unused platforms, and internal dataset labels are listed in Supplementary Table S13.

#### 2.5.4 Problematic use composite

The Problematic Use Composite is a *de novo* composite constructed for this study and is not a validated instrument. It comprises eight statements rated from 1 (strongly disagree) to 5 (strongly agree), three of which are worded in the direction of controlled use and are reverse-scored before summation, yielding a total score from 8 to 40, with higher values indicating more problematic use (observed range 8–40; mean 24.51, SD 6.95; Cronbach’s α = 0.807; item wording and item statistics in Supplementary Table S1). Because it is unvalidated, it was analyzed as a secondary, exploratory measure, was not used to define groups, and no clinical threshold was applied.

### 2.6 Outcome definitions

#### 2.6.1 Sleep quality

The primary sleep outcome was the PSQI global score, a continuous measure from 0 to 21 obtained by summing seven components (subjective quality, latency, duration, habitual efficiency, disturbances, medication use, and daytime dysfunction), each scored 0–3 according to Buysse et al. (34), with higher scores indicating poorer sleep quality. The secondary binary outcome classified participants as poor sleepers using a published cutoff of > 5. Habitual sleep efficiency was derived from reported bedtime, wake time, and actual sleep duration using the published algorithm. The PSQI was administered in English without translation, and English is the language of instruction and assessment throughout undergraduate medical and dental education in Pakistan. The item content, item order, and scoring algorithm were those of the published instrument. The response labels of the sleep disturbance grid, however, were administered as never/rarely (less than once a week)/sometimes (once or twice a week)/often (three to five times a week); the top category is narrower than the published “three or more times a week.” The four categories map one-to-one onto the published 0–3 scoring, and the components are scored identically; however, the instrument is accordingly described here as the PSQI with modified disturbance-grid labels rather than the unmodified original (see Section 4.9).

#### 2.6.2 Academic performance

The primary academic outcome was the self-reported percentage of marks obtained in the most recent professional examination. In Pakistani MBBS and BDS programs, these are summative, university-administered assessments taken at the end of each academic year, comprising written papers along with viva voce and objective structured clinical or practical examinations, marked on a 0–100 scale with a customary pass mark of 50%. This single annual percentage is the standard index of academic standing in this setting, as no grade point average is issued. Readers accustomed to GPA-based systems should consider it a year-level composite rather than a single-test score.

### 2.7 Ethical approval and consent

The protocol was approved by the Peshawar Medical College Ethical Review Committee (PRIME/ERC/2023-25), and the study was conducted in accordance with the Declaration of Helsinki. Because the design was anonymous, the Committee approved consent on an implied basis rather than through a written form. The questionnaire began with a participant information preamble that included the same information the data collector provided verbally (Section 2.4), along with a statement indicating that completing and returning the form constituted consent. No signature was collected, as a signed consent form would have been the only document capable of linking a participant to a returned questionnaire. Responses were stored in password-protected files accessible only to the research team, and only aggregated statistics were reported.

### 2.8 Statistical analysis

Analyses were conducted in Python 3.9.6, using NumPy and pandas for data handling, SciPy for non-parametric tests and distributional functions, and statsmodels for regression models, their diagnostics, and sensitivity analyses. Statsmodels does not implement Firth’s penalized likelihood, so the penalized logistic models in Supplementary Table S7 were fitted with a custom penalized iteratively reweighted least-squares (IRLS) routine written for this study, applying the Jeffreys-prior penalty of Firth (29) as described by Heinze and Schemper (30). Continuous variables are summarized as median (IQR), with mean (SD) also reported for the two outcomes to allow comparison with published literature; categorical variables are presented as counts and percentages.

Bivariate associations were examined with Spearman’s rank correlation, and group comparisons were made using the Kruskal–Wallis test (35) followed by pairwise Mann–Whitney U tests with Bonferroni adjustment. Categorical associations used Pearson’s chi-square without continuity correction, with Cramér’s V as the effect size. Cochran’s criterion was satisfied in every table, with a single cell having an expected count below five identified in Section 3.4. Non-parametric methods were used because several exposures were ordinal, and several continuous variables deviated from normality.

#### 2.8.1 Model specification

The conceptual framework specified before modeling (Supplementary Figure S1) informed the structure of the model. Sex, age (represented by year of study), program, institution type, and residence were included as background covariates plausibly related to both social media behavior and the outcomes; the five behavioral measures defined in Section 2.5.3 were the exposures of interest. Two specifications were reported (Supplementary Note S1): minimally adjusted models containing background covariates and mutually adjusted models additionally conditioning each exposure on the others. Both were interpreted as adjusted associations and were not claimed to identify total or direct causal effects. No stepwise or data-driven selection was performed, and the year of study was entered categorically, using the second year as the reference.

Multivariable linear regression was used for the PSQI global score and examination percentage, while multivariable logistic regression was used for poor sleep quality (PSQI > 5). Linear regression was retained despite the non-normality of several marginal distributions because its assumptions concern the residuals, which were assessed using the D’Agostino–Pearson K^2^ omnibus test for normality, the Breusch–Pagan test for homoscedasticity, variance inflation factors for collinearity, and leverage values for influence. Logistic model fit and discrimination were assessed using the Hosmer–Lemeshow test and the area under the ROC curve. Median (quantile) regression and bias-corrected bootstrap confidence intervals (2,000 resamples) were used for sensitivity analyses. In a secondary analysis, the academic time percentage (Q16) was added to the mutually adjusted models (Supplementary Table S8). The four ordinal exposures entered the primary models as numeric scores, assuming a constant mean outcome difference per one-category increase, despite unequal category widths. As a sensitivity analysis, they were refitted as categorical factors, with Q16 entered categorically in a separate model (Supplementary Table S9).

Whether MBBS and BDS students could be analyzed as a single cohort was tested rather than assumed by fitting program × exposure interaction terms for all behavioral exposures. Regression coefficients are reported with standard errors, 95% confidence intervals, standardized betas, *p*-values, and odds ratios with 95% confidence intervals for logistic models. All tests were two-sided at *p* < 0.05. No adjustment was made for multiplicity beyond the Bonferroni correction applied to the post-hoc pairwise comparisons: the two principal exposure findings have *p*-values well below conventional thresholds and are not materially affected by that choice, while all secondary, subgroup, and sensitivity results are interpreted as exploratory.

#### 2.8.2 Outcome specificity

Because the study’s central question is whether the two outcomes are associated with different features of use, the difference between the two models was tested directly rather than being inferred from a comparison of their statistical significance. Each participant contributed two records to a stacked dataset, one for each outcome. The PSQI global score and the reversed examination percentage were each standardized so that higher values indicated a worse outcome on both, and the four ordinal exposures were standardized so that the coefficients were standardized betas. A single model regressed the stacked outcome on the outcome type interacted with every term of the primary specification, with standard errors clustered on the participant, making the outcome × exposure interaction the standardized association with the reversed examination outcome minus that with sleep quality. A joint Wald test was applied to the two exposures carrying the principal findings, with Holm–Bonferroni adjustment across the four behavioral exposures reported alongside unadjusted values (Supplementary Table S10).

#### 2.8.3 Criterion overlap

The duration of pre-sleep social media use and three of the seven PSQI components (sleep latency, sleep duration, and habitual sleep efficiency) refer to the same interval of the night; therefore, an association between them could reflect definitional overlap rather than a substantive relationship. As a sensitivity analysis, the sleep model was refitted with those components removed from the outcome, and each component was modeled separately (Supplementary Table S11).

#### 2.8.4 Statistical power

Power was addressed *a priori* through the minimum detectable effects reported in Section 2.3, which were computed from the design rather than the results. The observed (post-hoc) power is not reported, as it is a deterministic function of the observed *p*-value.

## 3 Results

### 3.1 Sample characteristics

The analytical sample comprised 364 students (Table 1). The mean age was 22.09 years (SD 1.68; median 22, IQR 21–23, range 18–27), and 204 (56.0%) were male. Most were in clinical years (298, 81.9%), enrolled in MBBS programs (252, 69.2%), and attending private-sector institutions (259, 71.2%). No values were missing for any analytic variable.

**Table 1:** Characteristics of the study sample (*n* = 364).

Characteristic	Category	*n* (%)
Age, years	Median 22 (IQR 21–23); mean 22.09 (SD 1.68)	—
Sex	Male	204 (56.0)
	Female	160 (44.0)
Year of study	2nd year (pre-clinical)	66 (18.1)
	3rd year (clinical)	100 (27.5)
	4th year (clinical)	99 (27.2)
	Final year (clinical)	99 (27.2)
Program	MBBS	252 (69.2)
	BDS	112 (30.8)
Institution type	Private	259 (71.2)
	Public	105 (28.8)
Residence	Off-campus/with family	216 (59.3)
	Hostel/on-campus	148 (40.7)
Years using social media	More than 6 years	143 (39.3)
	4–6 years	140 (38.5)
	1–3 years	70 (19.2)
	Less than 1 year	11 (3.0)
Total daily social media use	More than 5 h	128 (35.2)
	3–5 h	78 (21.4)
	2–3 h	70 (19.2)
	1–2 h	62 (17.0)
	30 min – 1 h	18 (4.9)
	Less than 30 min	8 (2.2)
Purpose of use	Entertainment-dominated	210 (57.7)
	Academic	86 (23.6)
	Mixed	68 (18.7)
Study-time multitasking	Often	165 (45.3)
	Always	74 (20.3)
	Rarely	68 (18.7)
	Sometimes	57 (15.7)
Duration of pre-sleep social media use	30–60 min	179 (49.2)
	More than 60 min	89 (24.5)
	15–30 min	59 (16.2)
	Less than 15 min	37 (10.2)
Nocturnal checking	Sometimes	173 (47.5)
	Often	115 (31.6)
	Rarely	44 (12.1)
	Never	32 (8.8)
Phone location during sleep	Bedside table, screen-up	221 (60.7)
	In hand/under pillow	74 (20.3)
	In room, face-down/silent	69 (19.0)
Sleep quality (PSQI)	Poor (> 5)	312 (85.7)
	Good (≤ 5)	52 (14.3)
PSQI global score	Median 10 (IQR 8–13); mean 10.09 (SD 3.64)	—
Examination percentage	Median 77.0 (IQR 72.3–82.1); mean 77.29 (SD 6.78)	—

Mean (SD) is reported for the two outcome variables to facilitate comparison with published literature; all other continuous summaries are reported as median (IQR).

### 3.2 Patterns of social media use

Multi-platform use was nearly universal. A total of 363 out of 364 students (99.7%) used two or more platforms, with a mean of 4.87 platforms per student (SD 1.19; range, 1–8). YouTube was the most widely used platform (356, 97.8%), while Twitter/X was the least used (82, 22.5%). The most frequently reported primary purpose differed markedly by platform: Instagram, TikTok, and Snapchat were predominantly used for entertainment, LinkedIn for academic purposes, and the responses for YouTube, WhatsApp, Facebook, and Twitter/X were more dispersed (Table 2).

**Table 2:** Platform use and primary purpose among users (*n* = 364).

Platform	Users, *n* (%)	Most frequently reported primary purpose	*n*	% of users
YouTube	356 (97.8)	Academic/study	161	45.2
WhatsApp	353 (97.0)	Communication with peers	153	43.3
Instagram	302 (83.0)	Entertainment/leisure	206	68.2
TikTok	224 (61.5)	Entertainment/leisure	173	77.2
Facebook	198 (54.4)	Entertainment/leisure	74	37.4
Snapchat	143 (39.3)	Entertainment/leisure	94	65.7
LinkedIn	114 (31.3)	Academic/study	72	63.2
Twitter/X	82 (22.5)	News/information	47	57.3

Percentages of users are calculated within each platform’s user base. The table reports the most frequently reported primary purpose for each platform; full cross-tabulations are available in Supplementary Table S2.

Use was concentrated late in the day: 194 students (53.3%) reported peak use between 9 p.m. and midnight, and a further 107 (29.4%) after midnight. Pre-sleep use was nearly universal in some form, with 268 students (73.6%) reporting 30 min or more of use before sleep and 295 (81.0%) keeping their phone on the bedside table or in bed itself.

### 3.3 Sleep quality

Poor sleep quality (PSQI global score > 5) was observed in 312 students (85.7%, 95% CI 81.7–88.9). The median global score was 10 (IQR 8–13; range 2–18; mean 10.09, SD 3.64). The internal consistency across the seven components was acceptable (Cronbach’s α = 0.779).

The burden was distributed across most components rather than being confined to one. Sleep duration was the most affected, with 267 students (73.4%) scoring 2 or 3, followed by daytime dysfunction (255, 70.1%), sleep latency and sleep disturbance (both 186, 51.1%), subjective sleep quality (182, 50.0%), and habitual sleep efficiency (145, 39.8%). The median sleep latency was 20 min (IQR 15–30), the mean reported sleep duration was 5.23 h (SD 0.83), and 267 students (73.4%) slept for fewer than six hours. Habitual sleep efficiency ranged from 51.9 to 96.0% (mean 77.2%), with 77 students (21.2%) at or above the 85% threshold, scoring 0. Sleep medication was the only component rarely affected at the level of 2 or 3 (11, 3.0%), although 78 students (21.4%) reported using it in the past month.

### 3.4 Outcomes by purpose of use

Both outcomes differed across the purpose groups (Table 3). For the PSQI global score, H(2) = 10.22, *p* = 0.006, η^2^H = 0.023, with the academic group differing from the entertainment-dominated group (*p* = 0.005) but not from the mixed group; the mixed and entertainment groups did not differ. For examination percentage, the pattern was similar, H(2) = 11.46, *p* = 0.003, η^2^H = 0.026, again with only the academic and entertainment-dominated groups differing (*p* = 0.006).

**Table 3:** Outcomes by purpose of social media use (*n* = 364).

Purpose group	*n*	PSQI global, median (IQR)	Examination %, median (IQR)
Academic	86	9.0 (5.0–12.0)	78.8 (74.5–84.2)
Mixed	68	10.0 (7.8–13.0)	77.8 (74.6–82.2)
Entertainment-dominated	210	11.0 (8.0–13.0)	75.8 (71.9–80.7)
Kruskal–Wallis		H(2) = 10.22, *p* = 0.006, η^2^H = 0.023	H(2) = 11.46, *p* = 0.003, η^2^H = 0.026

Post-hoc comparisons were conducted using Mann–Whitney U tests with Bonferroni adjustment.

In unadjusted chi-square tests, poor sleep was associated with the duration of pre-sleep social media use (χ^2^(3) = 42.05, *p* < 0.001, Cramér’s V = 0.340), purpose of use (χ^2^(2) = 14.27, *p* < 0.001, V = 0.198), and study-time multitasking (χ^2^(3) = 12.79, *p* = 0.005, V = 0.187), but not with nocturnal checking (χ^2^(3) = 7.67, *p* = 0.053), year of study (*p* = 0.056), sex, program, institution type, or residence (all *p* > 0.11). Cochran’s criterion was satisfied throughout; the smallest expected count was 4.57 in the nocturnal checking table, and a Monte Carlo test with 20,000 permutations yielded the same conclusion (*p* = 0.054).

### 3.5 Bivariate associations

The Spearman correlations are presented in Table 4. Among the exposures, the duration of pre-sleep social media use showed the strongest association with the PSQI global score (ρ = 0.485), followed by problematic use severity (ρ = 0.440), total daily use (ρ = 0.360), study-time multitasking (ρ = 0.315), and nocturnal checking (ρ = 0.203), all *p* < 0.001. For examination percentage, the strongest correlates were problematic use severity (ρ = −0.530), academic time percentage (ρ = 0.506), total daily use (ρ = −0.441), and pre-sleep use (ρ = −0.386), all *p* < 0.001. Sleep quality and examination percentage were weakly correlated (ρ = −0.166, *p* = 0.001).

**Table 4:** Spearman correlations among exposures and outcomes (n = 364).

Variable	1	2	3	4	5	6	7	8	9
1 Total daily use	—								
2 Academic time %	−0.514***	—							
3 Study-time multitasking	0.519***	−0.389***	—						
4 Duration of pre-sleep social media use	0.629***	−0.417***	0.443***	—					
5 Nocturnal checking	0.306***	−0.304***	0.174***	0.364***	—				
6 Problematic Use Composite	0.718***	−0.636***	0.553***	0.691***	0.380***	—			
7 Number of platforms	0.324***	−0.155**	0.164**	0.290***	0.091	0.243***	—		
8 PSQI global score	0.360***	−0.282***	0.315***	0.485***	0.203***	0.440***	0.170**	—	
9 Examination percentage	−0.441***	0.506***	−0.319***	−0.386***	−0.231***	−0.530***	−0.108*	−0.166**	—

*p < 0.05, **p < 0.01, ***p < 0.001. Exact *p*-values are given in Supplementary Table S3.

Study-time multitasking and duration of pre-sleep social media use correlated moderately but remained distinct (ρ = 0.443, *p* < 0.001), and both were retained in all models.

### 3.6 Factors associated with sleep quality

The adjusted model explained 26.3% of the variance in the PSQI global score (F(13, 350) = 9.58, *p* < 0.001) (Figure 1A; Supplementary Table S4). One exposure was independently associated with poorer sleep, specifically the timing of use: duration of pre-sleep social media use (B = 1.594, 95% CI 1.081–2.106, *p* < 0.001). It had the largest standardized association in the model by a wide margin (β = 0.392, with the next largest behavioral coefficient being study-time multitasking at β = 0.077).

**Figure 1:**
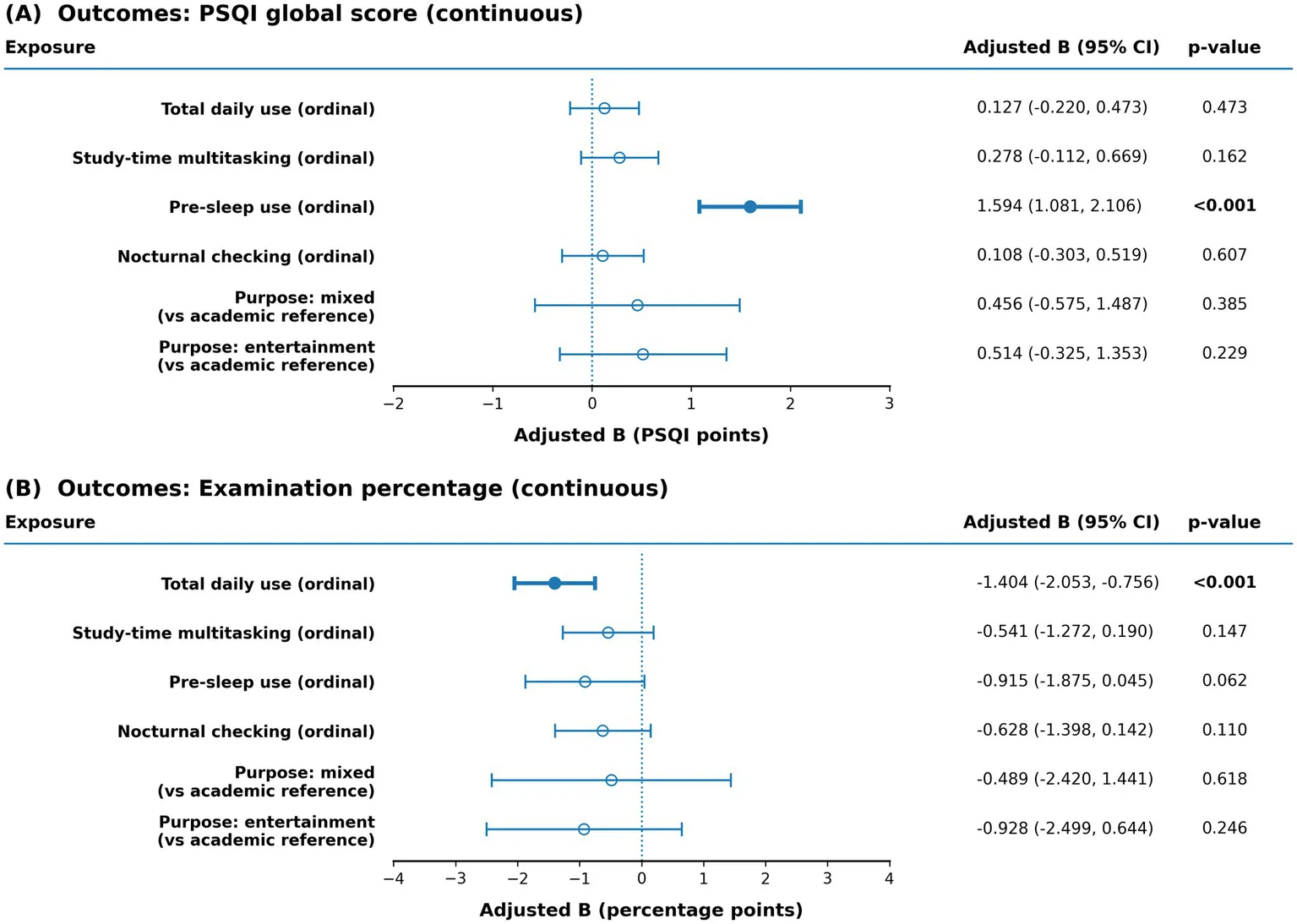
Adjusted associations of social media exposures with study outcomes. Points represent unstandardized regression coefficients from the mutually adjusted models, and horizontal lines indicate 95% confidence intervals with end caps. The numeric column to the right of each panel repeats the coefficient, interval, and *p*-value. Filled markers on thick lines denote *p* < 0.05, while open markers on thin lines denote *p* ≥ 0.05. **(A)** Shows the PSQI global score, where a higher score indicates poorer sleep; **(B)** illustrates the examination percentage, where a lower value indicates a lower mark. For the four ordinal exposures (total daily use, study-time multitasking, duration of pre-sleep social media use, and nocturnal checking), coefficients represent one-category increases. The two purpose coefficients are contrasts with academic purpose as the reference category, and “Purpose: entertainment” refers to the entertainment-dominated category defined in Section 2.5.3. The vertical dotted line marks the null. Both models adjust for sex, year of study, program, institution type, and residence, and are mutually adjusted for the other social media exposures. PSQI, Pittsburgh Sleep Quality Index; CI, confidence interval. Full coefficients, standard errors, standardized betas, and exact *p*-values are provided in Supplementary Tables S4, S5.

Nocturnal checking was not independently associated with the PSQI global score once pre-sleep duration was included (B = 0.108, 95% CI − 0.303 to 0.519, *p* = 0.607), despite its unadjusted correlation (Section 3.5). The same was true for study-time multitasking (B = 0.278, 95% CI − 0.112 to 0.669, *p* = 0.162). Neither total daily use (B = 0.127, 95% CI − 0.220 to 0.473) nor an entertainment-dominated purpose (B = 0.514, 95% CI − 0.325 to 1.353) was independently associated with poor sleep quality. Among the covariates, only third-year students differed from second-year students (B = 1.093, 95% CI 0.099 to 2.087, *p* = 0.031); the year-of-study pattern was not monotonic, and this contrast did not survive the clustering sensitivity analysis (Section 3.11).

### 3.7 Factors associated with academic performance

The adjusted model explained 25.4% of the variance in the examination percentage (F(13, 350) = 9.17, *p* < 0.001) (Figure 1B; Supplementary Table S5). In contrast to the sleep model, the independent association concerned the amount rather than the timing of use: each increment in the total daily use category was associated with examination scores 1.40 percentage points lower (B = −1.404, 95% CI − 2.053 to −0.756, *p* < 0.001, β = −0.282), the largest standardized association in the model. Under the linear-per-category specification, this implies a five-category contrast of approximately 7.0 percentage points between the lowest and highest use categories, which should not be interpreted as a literal contrast between numbers of hours due to the unequal category widths. MBBS students scored 1.57 percentage points higher than BDS students (95% CI 0.225 to 2.906, *p* = 0.022), a difference that did not survive the clustering sensitivity analysis and is discussed in Section 4.6 as a feature of the assessment scale rather than attainment.

Platform-derived purpose classification was not independently associated with examination percentage: relative to academic-purpose use, entertainment-dominated use was associated with scores 0.93 points lower (95% CI − 2.499 to 0.644), and mixed use with scores 0.49 points lower (95% CI − 2.420 to 1.441). Study-time multitasking (*p* = 0.147), pre-sleep use (*p* = 0.062), and nocturnal checking (*p* = 0.110) were also not independently associated with it. In a secondary model that additionally included academic-use proportion (Q16), each one-category increase was associated with a 2.148-percentage-point higher score (95% CI 1.513 to 2.782, *p* < 0.001), while total daily use remained associated with lower scores, with a smaller coefficient that did not survive the clustering sensitivity analysis (B = −0.809, 95% CI − 1.445 to −0.172, *p* = 0.013); model R^2^ rose to 0.338. The academic-use proportion was not associated with the PSQI global score (*p* = 0.198); the full coefficients are presented in Supplementary Table S8.

### 3.8 Poor sleep quality as a binary outcome

The adjusted logistic model discriminated acceptably (Table 5): LR χ^2^(13) = 53.52, *p* < 0.001; Nagelkerke R^2^ = 0.244; area under the ROC curve = 0.800; Hosmer–Lemeshow χ^2^(8) = 9.64, *p* = 0.292. The principal finding of the linear model was reproduced: each one-category increase in pre-sleep social media use was associated with 2.70 times the odds of poor sleep (95% CI 1.66–4.38, *p* < 0.001). The two predictors differed between the specifications. Entertainment-dominated purpose, which was not associated with the continuous score, carried higher odds of the binary outcome relative to academic-purpose use (aOR 2.58, 95% CI 1.22–5.43, *p* = 0.013; not robust to the clustering sensitivity analysis), with mixed purpose close to the conventional threshold (aOR 2.66, 95% CI 0.97–7.30); and third-year students had higher odds than second-year students (aOR 2.55, 95% CI 1.01–6.48, *p* = 0.048). Neither nocturnal checking, study-time multitasking, nor total daily use was associated with the binary outcome (all aOR ≈ 1.0, *p* > 0.84). Because dichotomization discards information and the outcome is heavily unbalanced (Section 2.3), the continuous model was the primary analysis, and the purpose of the association here was interpreted as exploratory.

**Table 5:** Logistic regression model for poor sleep quality (*n* = 364).

Predictor	aOR	95% CI	*p*
Male sex	1.15	0.59–2.22	0.684
3rd year	2.55	1.01–6.48	0.048
4th year	2.50	0.94–6.65	0.067
Final year	1.67	0.69–4.07	0.255
MBBS program	1.13	0.56–2.29	0.724
Private institution	0.58	0.27–1.25	0.166
Hostel/on-campus residence	1.40	0.70–2.80	0.342
Total daily social media use	0.97	0.68–1.37	0.844
Purpose: mixed	2.66	0.97–7.30	0.057
Purpose: entertainment-dominated	2.58	1.22–5.43	0.013
Study-time multitasking	0.99	0.63–1.53	0.949
Duration of pre-sleep social media use	2.70	1.66–4.38	< 0.001
Nocturnal checking	1.03	0.72–1.48	0.873

LR χ^2^(13) = 53.52, *p* < 0.001; Nagelkerke R^2^ = 0.244; AUC = 0.800; Hosmer–Lemeshow χ^2^(8) = 9.64, *p* = 0.292. Reference categories: female, 2nd year, BDS, public institution, off-campus residence, academic purpose.

### 3.9 Minimally and mutually adjusted associations of purpose of use

Under the minimally adjusted specification, entertainment-dominated use was associated with poorer sleep quality (B = 1.453, 95% CI 0.541 to 2.364, *p* = 0.002) and lower examination percentage (B = −2.835, 95% CI − 4.522 to −1.148, *p* = 0.001). Both were substantially attenuated in the mutually adjusted models in Sections 3.6 and 3.7, in which the three-category classification had no independent association with either outcome. This is compatible with purpose associations overlapping with correlated differences in when and how much students use social media, although the design does not identify mediation effects. The residual association with the binary sleep outcome (Section 3.8) is the exception and should be weighed against the null result for the continuous score in the same participants.

### 3.10 Incremental value of purpose and timing beyond total use

Adding the purpose of use, study-time multitasking, pre-sleep use, and nocturnal checking to a model containing the demographic covariates and total daily use increased explained variance from 15.6 to 26.3% for the PSQI global score (ΔR^2^ = 0.107; F(5, 350) = 10.11, *p* < 0.001) but only from 22.6 to 25.4% for examination percentage (ΔR^2^ = 0.028; F(5, 350) = 2.64, *p* = 0.023). Therefore, the four additional variables significantly contributed to sleep quality but had little impact on academic performance, where total daily use carried most of the signal.

### 3.11 Model diagnostics and sensitivity analyses

The regression diagnostics were satisfactory. Residuals were normally distributed for both models (PSQI: D’Agostino–Pearson K^2^ = 1.46, *p* = 0.483; examination: K^2^ = 1.52, *p* = 0.468) and homoscedastic (Breusch–Pagan *p* = 0.710 and 0.718, respectively). All variance inflation factors were ≤ 2.06, and the maximum leverage was 0.072.

Both principal findings survived all specification checks. Median regression reproduced them (pre-sleep use with PSQI B = 1.679, 95% CI 1.061 to 2.297; total daily use with examination percentage B = −1.833, 95% CI − 2.626 to −1.040, both *p* < 0.001), as did the categorical coding of the four ordinal exposures (F(3, 340) = 11.64 and F(5, 340) = 3.49 respectively, both *p* ≤ 0.004). The sleep model was refitted on restricted outcomes to address criterion overlap, where the association persisted across the three PSQI components with no definitional overlap with the exposure: sleep disturbance, medication use, and daytime dysfunction, with standardized β declining from 0.392 to 0.257. Bias-corrected bootstrap intervals agreed with ordinary least squares. Some secondary coefficients were specification-dependent: study-time multitasking became associated with PSQI under categorical coding (*p* = 0.039) but was borderline in median regression, and third year and MBBS lost significance in the median regression. The categorical models did not improve the fit over the linear-per-category specifications (PSQI *p* = 0.154; examination *p* = 0.901); therefore, the primary coding was retained. The program × exposure interaction terms were non-significant for both outcomes (PSQI F(6, 344) = 0.457, *p* = 0.840; examination F(6, 344) = 1.716, *p* = 0.116), supporting the analysis of MBBS and BDS students as a single cohort. The full results are presented in Supplementary Tables S6, S9, S11.

Because participants were clustered within ten institutions and the institution identifier was not recorded, the standard error of every reported estimate was inflated by √DEFF under assumed intracluster correlations of 0.01 and 0.02, as outlined in Section 2.3 (Supplementary Table S12). This is an arithmetic sensitivity analysis of the reported estimates, not a refitted multilevel model, and the two values of ρ are assumed rather than estimated. Both principal findings were retained under the more conservative assumption (ρ = 0.02): pre-sleep use with the PSQI global score (widened 95% CI 0.924 to 2.264, *p* < 0.001) and total daily use with examination percentage (−2.252 to −0.556, *p* = 0.001), as were the academic-use proportion in the secondary model (1.319 to 2.977) and pre-sleep use in the logistic model (aOR 1.43 to 5.09). Four smaller estimates did not: the third-year contrast for PSQI (−0.206 to 2.392), the MBBS–BDS difference in examination percentage (−0.186 to 3.318), the entertainment-dominated contrast for the binary sleep outcome (aOR 0.97 to 6.84), and total daily use in the secondary examination model (−1.641 to 0.023). Of the two outcome-specificity contrasts (Section 3.12), the pre-sleep contrast was retained (−0.522 to −0.020, *p* = 0.034), while the total-use contrast was not (−0.030 to 0.500, *p* = 0.082). Only associations surviving both scenarios were carried forward as findings.

### 3.12 Outcome specificity of the exposure associations

The two principal findings were compared directly in a single model fitted to both outcomes simultaneously (Section 2.8; Supplementary Table S10). The association of pre-sleep social media use with sleep quality was stronger than its association with examination performance (difference in standardized β = −0.271, 95% CI − 0.463 to −0.079, *p* = 0.006), and the association of total daily use with examination performance was stronger than its association with sleep quality (difference = 0.235, 95% CI 0.032 to 0.437, *p* = 0.023). Neither study-time multitasking (*p* = 0.966) nor nocturnal checking (*p* = 0.429) differed between the outcomes, nor did either purpose contrast. A joint Wald test of the two exposures carrying the principal findings was significant (χ^2^(2) = 8.30, *p* = 0.016). Under the Holm–Bonferroni adjustment, the pre-sleep contrast remained significant (adjusted *p* = 0.023), while the total-use contrast was attenuated to *p* = 0.069, and the clustering sensitivity analysis ordered them the same way (Section 3.11). Therefore, the case for outcome specificity rests principally on pre-sleep use.

## 4 Discussion

### 4.1 Principal findings

In these students, the primary models associated poorer sleep with one aspect of usage timing, specifically the duration of use in the hour before sleep, and lower examination marks with the total amount of daily use. Nocturnal checking and platform-derived purpose classification were independently associated with neither once amount and timing were included. A secondary measure, the proportion of use devoted to academic work, was associated with higher examination scores. Two points frame the following discussion. Fitting both outcomes in a single model showed that the two principal associations differed between outcomes rather than merely differing in statistical significance (Section 3.12); however, median regression reproduced only those two findings, so the outcome-specific pattern should not be interpreted as an absolute absence of cross-outcome associations.

### 4.2 Timing of use and sleep quality

The duration of pre-sleep social media use was the strongest correlate of poor sleep in this sample in both the continuous and binary analyses and had the largest standardized association in the adjusted model. This is consistent with evidence that the timing of use within the day is more important than its total volume. In an intensive app-tracking study of adolescents, only post-bedtime use was associated with sleep quality, while daytime use was not (36). Nocturnal checking behavior was different. It correlated with the unadjusted PSQI global score (ρ = 0.203) but provided no independent information once pre-sleep duration was included. The likely interpretation is shared variance: checking correlated with pre-sleep duration (ρ = 0.364), and the null result was reproduced in the logistic model, median regression, and under categorical coding (Section 3.11), which fits an absent independent association better than a specification artifact. Checking frequency has been reported to be more strongly associated with sleep disturbance than duration (37), nocturnal awakenings involving phones have been prospectively linked to restless sleep (38), and objectively recorded phone use after sleep onset has been associated with poor sleep and lower well-being (39). Our data are compatible with those reports at the bivariate level but do not support an independent contribution beyond pre-sleep use. Shared-method bias also had to be considered because the exposure and three of the seven PSQI components refer to the same interval of the night, but it did not account for the finding: the association survived the removal of those components and persisted on the three with no definitional overlap (β 0.392 to 0.257; Section 3.11).

The direction of this association cannot be resolved by these data, and noting that as a limitation understates what is at stake because the two directions imply different interventions. In the forward pathway, engagement in the hour before sleep raises cognitive and physiological arousal at the point when disengagement is required: experimentally induced pre-sleep cognitive arousal prolongs sleep-onset latency (40), the hyperarousal model of insomnia describes the resulting state as activation persisting into the sleep-onset period (41), and screen light close to bedtime has been proposed to suppress melatonin and delay circadian phase, with bedtime use of blue-light-emitting devices linked to perceived sleep disturbance among medical students (42). In the reverse pathway, arousal comes first, followed by the phone. A student already lying awake, whether from examination anxiety, an irregular schedule, or an undiagnosed sleep disorder, has both the opportunity and the motive to reach for a device, and social media then serves as an emotion-regulation or distraction strategy for insomnia that it did not create; the bidirectional model of sleep and technology use accounts for this (23). Two features of these data bear on the distinction without resolving it. The item “I check social media as a way to cope with academic stress” was endorsed at a mean of 3.24 on a five-point scale (Supplementary Table S1), indicating that a coping motive is common. In contrast, nocturnal checking, which is the behavior most indicative of already being awake at night and the one a coping account would predict to carry the strongest association, is precisely the exposure that diminished upon adjustment. Therefore, we consider both pathways as active and likely concurrent and make no claim to have identified a direction; separating them requires intensive longitudinal measurement in which within-person pre-sleep use and same-night sleep can be ordered in time or through experimental restriction of pre-sleep device availability (Section 4.10).

Whatever the direction, the magnitude should be compared against the largest available datasets, which point the other way: in the UK Millennium Cohort data on 11,884 adolescents, associations between overall digital engagement and sleep were very small. An additional hour of daily screen time corresponded to approximately nine fewer minutes of weekday sleep, and use before bedtime was not substantively associated with sleep duration or bedtime (43). Three differences may explain the discrepancy: a validated multi-component sleep instrument as the outcome rather than a single sleep measure, a high-burden sample averaging 5.23 h of sleep and therefore offering more variation to explain, and an undergraduate professional program rather than a school-aged population. A fourth consideration cuts the other way since exposure and outcome were reported by the same respondent on the same instrument (Section 4.9). The direction of our findings is consistent with the literature, and the magnitude is likely to be an upper bound.

Study-time multitasking was not independently associated with sleep once pre-sleep duration and nocturnal checking were included in the model, despite a moderate unadjusted correlation (ρ = 0.315). However, it did reach significance under categorical coding (Section 3.11), indicating that this null result is specification-dependent rather than strong evidence of no association. The most straightforward interpretation is that multitasking characterizes students whose usage extends into the evening rather than being a sleep behavior in its own right; it correlated moderately with pre-sleep duration (ρ = 0.443) and total daily use (ρ = 0.519). One important qualification is that academically relevant multitasking has been found to correlate positively with self-regulation, flow, and achievement, while irrelevant multitasking showed no such association (44). Our single item cannot separate these types; therefore, it may combine behaviors with opposing associations and attenuate any true effect. Self-regulatory failure during study remains a plausible mechanism within social cognitive theory (45, 46), but these data do not support it, and we do not claim it.

### 4.3 Amount of use and academic performance

Among the five primary behavioral exposures, total daily use was the only one independently associated with examination performance, and the association was graded across the exposure scale: each step up the six bands corresponded to 1.40 percentage points, a model-implied 7.0-point contrast across the five steps from the lowest to the highest category. Because the bands have unequal widths, this is not a literal per-hour contrast. A difference of that size would be of educational interest if it were proven causal, although this design cannot establish that.

Two interpretations are available, and the design cannot separate them. The first is displacement: hours spent on social media are hours not spent studying, with the association operating through time rather than the content or context of use. This aligns with the incremental-variance result, as adding purpose, multitasking, and the two timing variables to a model already containing total daily use raised explained variance by only 2.8 pp. for examination performance compared to 10.7 for sleep. The second is reverse causation: students struggling academically or less engaged with their program may have more unstructured time and use it for online activities. This association cannot be separated from academic self-concept or motivation, neither of which was measured. The broader evidence is mixed: AlFaris et al. found no significant association between GPA and the frequency of daily use or use during lectures (47), whereas smartphone addiction has been associated with lower academic performance among medical students (48). Students have described using social media to pass the time while regarding it as an interference with learning (49), and among dental students, smartphone addiction has been associated with a lower GPA (50), while social media use has also been reported to bring educational benefits alongside perceived drawbacks (51).

This relates to one strand of recent literature. The systematic review mentioned in the Introduction excluded studies that measured exposure solely as time spent or frequency of use, because these do not adequately capture problematic use as a construct (22). This is a definition of scope rather than a claim that time spent carries no information about achievement. Our data indicate that it does, as time spent was the only primary exposure to survive adjustment for this outcome; the review’s own pooled association was negative but not statistically significant (r = −0.114, 95% CI − 0.283 to 0.054), with substantial heterogeneity. Two qualifications apply: a self-estimated banded measure is an imperfect proxy for logged use (52), with consequences outlined in Section 4.9, and it may also reflect a general disposition toward heavy engagement that correlates with academic difficulty for reasons unrelated to time spent.

### 4.4 Purpose is associated with examination performance only when it is measured as time allocation

The platform-derived purpose classification was associated with both outcomes in the unadjusted analysis. Entertainment-dominated users experienced poorer sleep and scored lower, but neither effect was evident in the primary linear models, with substantial attenuation in both cases (Section 3.9). It retained an independent association only with the dichotomized sleep outcome (aOR 2.58), in an analysis involving 52 good sleepers and 4.0 events per variable, which was imprecise, not robust to the clustering sensitivity analysis, and not reproduced in the better-powered continuous model using the same participants; we do not regard this as evidence of an independent association. Students characterizing their use as entertainment-dominated also spent more time on social media and used it later, consistent with the overlap of the purpose association and those correlated behaviors. In contrast, the secondary academic-use-proportion measure was independently associated with examination percentages; therefore, conclusions about purpose depend on how it is operationalized.

This is a negative result for a measure, not for the concept. Purpose was self-classified from per-platform responses, and students who perform well may be more inclined to characterize their use as academic, so the variable is entangled with academic self-concept. It is also coarse: a student who identifies YouTube as academic may be watching lecture videos or entertainment content instead of reading. Uses and Gratifications Theory (53, 54) remains a plausible explanation for why students select particular media, and our data do not refute it; they only show that this three-category variable adds no independent information once the amount and timing are known. The self-concept caveat applies with at least equal force to the academic-use-proportion measure, which is also self-reported and carries a larger coefficient than the other two. Measures validated against content-level device data are needed to properly test the construct.

### 4.5 Prevalence of poor sleep quality in context

In this sample, 85.7% of students met the PSQI threshold for poor sleep quality. This exceeds the 59.1% pooled prevalence reported across 16 Middle Eastern and North African countries (2) and is higher than earlier international pooled estimates (1). It also surpasses the 48.3% reported among Indian medical undergraduates (55), the 63.7% among Indian dental undergraduates (56), and the 35.3% among Moroccan medical students, 97.3% of whom used a blue-light-emitting device at bedtime (42). Nighttime screen use has also been associated with higher PSQI scores among medical students (19). Therefore, the present estimate is higher than that of every published comparator we identified, which warrants caution rather than emphasis. The same MENA meta-analysis found no difference in poor sleep quality by academic performance (2), consistent with the dissociation reported in this study.

These comparisons require caution because PSQI prevalence is sensitive to sample composition and examination proximity, and a convenience sample cannot support a population prevalence claim. The component profile was nevertheless informative: sleep duration was the most affected component, followed by daytime dysfunction. For the four out of five students below the 85% efficiency threshold, the gap between time in bed and reported sleep was compatible with lying awake after going to bed and with the pre-sleep use finding. This pattern suggests insufficient and delayed sleep but does not exclude undiagnosed sleep disorders, which were not assessed.

### 4.6 Platform patterns, demographic factors, and the structure of the associations

Platform-level findings were less clear-cut than the purpose analysis suggested. Only four of the eight platforms had a purpose endorsed by 60% or more of their users: Instagram, TikTok, and Snapchat for entertainment, and LinkedIn for academic use. YouTube, the most widely used platform (97.8%), was divided almost evenly between academic (45.2%) and entertainment (43.5%) use, while WhatsApp was split between communication (43.3%) and academic use (38.2%). Platform identity is therefore a poor proxy for what a student is doing, which aligns with the finding that the number of platforms used is only weakly related to the PSQI global score (ρ = 0.170) and examination performance (ρ = −0.108). The established roles of WhatsApp as an informal educational tool (57) and YouTube as a video self-study resource (58) are evident in these data but not dominant in the results. Problematic TikTok use has been associated with shorter nocturnal sleep (59), and image-based platform use has been linked to poorer PSQI scores (60).

Demographic and structural characteristics were largely uninformative: sex, institution type, and residence were unrelated to both outcomes after adjustment. The absence of a sex difference is notable given reports that female students often report poorer sleep (10) and may indicate that the behavioral exposures absorb variance otherwise attributed to sex or may reflect limited power. Third-year students had higher PSQI global scores than pre-clinical students and higher odds of poor sleep, which may be related to the transition into clinical rotations. However, the contrast was marginal in both models, did not survive median regression or clustering sensitivity analysis (Section 3.11), was not consistent across years, and should not be over-interpreted.

The MBBS–BDS comparison requires more nuance than a single coefficient. MBBS students scored 1.57 percentage points higher than BDS students after adjustment, but that estimate did not survive the clustering sensitivity analysis (Section 3.11) and cannot be interpreted as a difference in attainment because the two programs sit for different examinations set and marked by different faculties. The programs also differ in curriculum and clinical exposure in ways that could plausibly shape digital routines: BDS training concentrates clinical work in dental clinics on comparatively fixed session timetables, whereas MBBS training rotates students through hospital wards and specialty attachments. Neither the timetable nor the clinical workload was recorded; therefore, these differences could not be directly modeled. However, whether they translated into different exposure–outcome relationships was tested, rather than assumed. The interaction terms were jointly non-significant for both outcomes, and the stratified models reproduced both principal associations in each stratum with the same sign, differing in precision rather than direction, which the smaller BDS subgroup (*n* = 112) would produce in the absence of effect modification (Section 3.11; Supplementary Table S6). Two qualifications are required. The interaction tests were not separately powered, so they show that these data provide no evidence of program-specific associations, not that none exists; at ΔR^2^ of 0.006 for the PSQI global score and 0.022 for examination percentage, a modest difference would have gone undetected. Nocturnal checking was associated with examination percentage in MBBS (B = −1.268, 95% CI − 2.174 to −0.362) but not in BDS (B = 1.193, 95% CI − 0.408 to 2.795), an opposition of sign that the joint test does not support and that we report for transparency rather than interpretation.

### 4.7 What this study adds

The individual exposures examined in this study are not novel. The timing of use, nocturnal checking, purpose of use, and total daily use have each been related to sleep or academic outcomes, including in multivariable models and in medical student populations (16–22). Any claim to have discovered that bedtime use disturbs sleep or that heavy use accompanies lower marks would be unsupportable. The closest precedent is an international survey of medical students in which screen use after nocturnal awakenings remained independently associated with poor sleep, while smartphone addiction symptoms did not (19): the same population, a comparable instrument, and the same conclusion structure for the nocturnal limb of our analysis.

However, we have not found a study that places all three exposure dimensions against both outcomes within the same adjusted model. This yielded an outcome-specific pattern. Pre-sleep timing was associated with sleep quality and not examination marks, while the amount of use was associated with examination marks and not sleep quality. These relationships were tested directly rather than inferred: in a model fitted to both outcomes simultaneously, the two associations differed significantly (Section 3.12), which is the comparison a claim of outcome specificity requires and which comparing significance across separate models cannot supply. The two outcomes most often reported together in this field therefore appear to relate to different features of the same behavior, with the practical implication that an intervention aimed at sleep and one aimed at academic performance may require different targets. The sample also addresses a gap, since dental undergraduates are seldom studied alongside medical students, and we found no comparable data from Khyber Pakhtunkhwa. These remain exploratory and cross-sectional associations that require replication.

### 4.8 Implications for public health practice

These findings generate outcome-specific hypotheses for prospective evaluation rather than establishing intervention effects. For sleep, the hypothesis is that reducing social media use in the hour before sleep—through a defined pre-sleep window or by keeping the phone out of the bed—would improve sleep quality even without reducing total daily use. For academic performance, the corresponding hypothesis is that reducing total daily use is more relevant than modifying bedtime use alone. Both should be tested prospectively or experimentally before being incorporated into institutional guidelines.

This setting makes such an evaluation relevant. Undergraduate training in Pakistan is examination-driven and residential for many students; 275 students (75.5%) indicated that they would likely or definitely join a structured digital well-being program if their institution offered one, while 167 (45.9%) reported that their college provided no guidance on healthy digital use or that they were unaware of any. Sleep health is not a routine component of undergraduate health professions curricula, and the burden documented above justifies prospectively evaluating such programs. One qualification applies to these targets: they are behavioral and are influenced by the institutional conditions that shape them, as the same institutions set the examination and workload structures under which a sample averaging 5.23 h of sleep was generated. Measures focused solely on student behavior are unlikely to be sufficient if curricular and assessment loads are not examined.

### 4.9 Limitations

#### 4.9.1 Cross-sectional design

Temporal order cannot be established; therefore, the direction of every association reported here remains unclear. The two candidate directions for the sleep finding and what in these data relates to each are set out in Section 4.2 above.

#### 4.9.2 Self-reported academic performance

Examination percentages were not verified against institutional records. Self-reported marks are typically inflated relative to transcripts, especially among lower-achieving students, which compresses the range and weakens associations. Recall error is an additional concern. The questionnaire also measured current or typical behavior, whereas the outcome was the most recent professional examination, which may have occurred earlier in the academic cycle. This temporal mismatch means that we cannot establish whether the reported behavior preceded the examination, which poses a principal threat to the internal validity of the academic analysis. Cross-institution comparability is another limitation, since seven of the ten colleges share an examining body while three do not, making absolute marks potentially non-comparable, and the absence of an institution identifier prevented adjustment for the examining body. No verification was possible under the anonymous design required for ethical approval.

#### 4.9.3 Self-reported exposure and sleep

All social media use was self-estimated, and self-reported media use correlated only moderately with device-logged use across 106 effect sizes (52); the banded total-use item was the most affected by this. Sleep was assessed using a questionnaire alone, whereas actigraphy or polysomnography would permit cross-validation. Two mechanisms operate that do not point in the same direction. Recall error is likely to be largely non-differential with respect to the outcomes, and non-differential misclassification of an ordinal exposure attenuates the coefficient toward the null, suggesting that the reported associations may understate the true association. Social desirability may be differentially influential: data collectors were peers from the participants’ own institutions, which may have encouraged under-reporting of heavy or late use and over-reporting of marks. Since the same respondent provided both, a student who understates use and overstates marks generates a spurious inverse association in precisely the direction reported. Therefore, information bias in either direction is possible, and its net direction cannot be determined here, which is why we describe these magnitudes as an upper bound (Section 4.2).

#### 4.9.4 Instrument validation

The social media items were developed for this study and have not undergone formal validation, so their construct validity is not established. The three-category purpose variable relied on participants’ own interpretation of their motivations and was independently associated with neither outcome in the primary linear models. This may reflect the crudeness of the measure as much as the absence of an association; the single multitasking item does not distinguish between academically relevant and irrelevant multitasking, which behaves differently (44). The PSQI was administered in English and has not been formally validated in an Urdu- or Pashto-speaking Pakistani population, and the response labels of the sleep disturbance grid differed from the published wording (Section 2.6); therefore, that component is not strictly comparable with studies using the original labels. Internal consistency was acceptable but modest (α = 0.779).

#### 4.9.5 Sampling and selection biases

Convenience recruitment with non-proportional site quotas means that the achieved sample reflects those who were accessible rather than a random draw, and that this is not a probability sample of medical and dental students in Khyber Pakhtunkhwa, let alone Pakistan. Therefore, selection bias is possible in both the prevalence estimates and exposure–outcome associations. Students who were absent, on placement away from the main campus, or less willing to disclose their habits were missing from the sample, and if their behavior, sleep, or attainment differed systematically from that of participants, both the 85.7% prevalence and the regression coefficients were affected in an unknown direction. The sampling fraction was approximately 5.5%, non-participant characteristics were unknown, and recruitment records were maintained at the study level rather than by institution; therefore, whether non-participation clustered at particular sites could not be determined. Data were entered once rather than in duplicate; therefore, the keystroke error rate could not be quantified. Every estimate should be interpreted as an association observed within the ten participating institutions.

#### 4.9.6 Clustering and the absence of institution-level data

Three distinct pieces of information were lost when the institution of enrollment was not recorded. The first is statistical: participants within a college share timetables, examination schedules, hostel arrangements, and peer norms; therefore, their responses are not independent, and treating them as independent results in standard errors that are too small. At intracluster correlations of 0.01 and 0.02, the intervals reported here are 16 and 31% too narrow (Section 2.3), and Section 3.11 identifies which associations survive this widening. The second is substantive: institution-level characteristics such as curricular intensity, examining body, timetabling, device policy, and campus connectivity are entirely unobserved, making it impossible to separate compositional variation from contextual variation. Any institution-level contribution is absorbed into the residual or misattributed to individual-level predictors correlated with the site; only public versus private sector was recorded, which serves as a weak proxy for the rest. The third is epistemic, which is why neither of the first two can be repaired by analysis: because the intracluster correlation cannot be identified from these data, the sensitivity analysis is conditional on assumed values and establishes a lower bound on the true uncertainty, rather than an upper one. We can say that the intervals are too narrow, but not by how much. The identifier was omitted at the questionnaire design stage and could not be recovered afterward because forms were deliberately pooled before numbering (Section 2.4); recording a non-identifying site code is the first design amendment we would make in any replication.

#### 4.9.7 Generalizability

All participants were drawn from a single province, and extending the findings to other low- and middle-income settings requires caution.

#### 4.9.8 Unmeasured confounding

Depression, anxiety, caffeine and other stimulant use, clinical duties, commuting time, and pre-existing sleep disorders were not assessed, and each is plausibly associated with both social media behavior and the outcomes; their omission is the most likely source of residual confounding. Depressive and anxiety symptoms are particularly consequential; they are associated with poorer sleep, lower attainment, and heavier and later use, suggesting they may serve as a common cause for both principal associations. If they act as one, the coefficients for pre-sleep use and total daily use are both biased away from the null, indicating that the true associations are smaller than reported; that bias cannot be bounded since neither construct was measured. Caffeine and stimulant use is a plausible confounder for the sleep model, likely biasing in the same direction. Clinical duties behave differently: undergraduate attachments here are timetabled rather than rostered as overnight calls, and their intensity is partly captured by the year of study, which was included in every model; what remains unobserved is workload variation between colleges and specialty attachments, which is missing for the same reason as the institution identifier. Pre-existing sleep disorders would raise the PSQI global score independently of behavior. Therefore, these models should be interpreted as associations adjusted for the measured covariates, rather than as approximations of the total or direct causal effect.

### 4.10 Future directions

Five priorities follow. First, prospective and experimental designs are needed to establish temporal order; a pilot trial restricting phone availability in the hour before sleep would directly test the strongest association reported here. Second, device-level logging and actigraphy address self-report and common method limitations, particularly regarding total daily use. Third, FoMO and self-regulatory capacity are plausible mediators of the timing–sleep association but were not measured. Fourth, multi-institutional studies using probability sampling and verified academic records should establish whether these associations hold in a representative sample. Fifth, replications should record the institution of enrollment as a non-identifying site code so that cluster-robust or multilevel estimation becomes possible and should record the curricular and clinical timetable along with the behavioral exposures.

## 5 Conclusion

Poor sleep quality was present in 85.7% of the 364 students sampled across ten institutions in Khyber Pakhtunkhwa (95% CI 81.7–88.9), and nearly three in four slept fewer than six hours. In the primary models, sleep quality was chiefly associated with the timing of use (the duration of use in the hour before bed), whereas examination marks were associated with total daily use; nocturnal checking was independently associated with neither. The platform-derived purpose classification was not associated with either outcome once the amount and timing were accounted for, whereas a secondary measure of academic-use proportion was associated with higher marks. A model fitted to both outcomes simultaneously indicated that the two principal associations differed between outcomes, most robustly for pre-sleep use, and both were retained when standard errors were inflated for plausible clustering by institution, whereas several smaller estimates were not retained. Within the limits of a cross-sectional convenience sample drawn from ten institutions, sleep and academic performance appear to relate to different aspects of social media behavior, and conclusions about purpose depend on how it is measured. The outcome-specific pattern requires replication with prospective designs, objective measures of use, and verified academic records before it can guide practice.

## Data Availability

The raw data supporting the conclusions of this article will be made available by the authors, without undue reservation.

## Glossary

aOR: adjusted odds ratio
AUC: area under the receiver operating characteristic curve
B: unstandardized regression coefficient
BDS: Bachelor of Dental Surgery
CI: confidence interval
df: degrees of freedom
IQR: interquartile range
IRLS: iteratively reweighted least squares
MBBS: Bachelor of Medicine, Bachelor of Surgery
PSQI: Pittsburgh Sleep Quality Index
PUC: Problematic Use Composite
R^2^: coefficient of determination
SD: standard deviation
SE: standard error
STROBE: Strengthening the Reporting of Observational Studies in Epidemiology
UGT: Uses and Gratifications Theory
VIF: variance inflation factor
β: standardized regression coefficient
η^2^H: eta-squared for the Kruskal–Wallis test
ρ: Spearman rank correlation coefficient
χ^2^: chi-square statistic
