# Supplementary material for: Differential associations of the timing and amount of social media use with sleep quality and academic performance among undergraduate medical and dental students in Pakistan: a cross-sectional study
Source: Front Public Health. 2026 Sep 28;14:1968305. doi: 10.3389/fpubh.2026.1968305 (PMC13655044; doi:10.3389/fpubh.2026.1968305)
Supplement: Supplementary file 1 [file Data_Sheet_1.docx]

# Differential Associations of the Timing and Amount of Social Media Use with Sleep Quality and Academic Performance Among Undergraduate Medical and Dental Students in Pakistan: A Cross-Sectional Study

# Supplementary Material

- Supplementary Table S1. Problematic Use Composite item statistics
- Supplementary Figure S1. Conceptual framework diagram informing covariate selection
- Supplementary Note S1. Model specifications reported in this article
- Supplementary Table S2. Platform-by-purpose cross-tabulation
- Supplementary Table S3. Spearman correlation p-value matrix
- Supplementary Table S4. Linear regression model for the PSQI global score
- Supplementary Table S5. Linear regression model for examination percentage
- Supplementary Table S6. Program-stratified linear regression models
- Supplementary Table S7. Maximum-likelihood and Firth penalized-likelihood logistic regression
- Supplementary Table S8. Secondary linear regression models including academic-use proportion (Q16)
- Supplementary Table S9. Sensitivity analyses treating ordinal exposures as categorical factors
- Supplementary Table S10. Outcome specificity: difference between the sleep and academic associations
- Supplementary Table S11. Criterion-overlap sensitivity analyses for duration of pre-sleep social-media use
- Supplementary Table S12. Clustering sensitivity analysis: estimates with standard errors inflated by the design effect
- Supplementary Table S13. Questionnaire items, response options and coding for every variable analyzed
- Supplementary Note S2. Sample size, analytic adequacy and data handling (supporting calculations)
- Supplementary Data Sheet 1. STROBE checklist for cross-sectional studies

**Supplementary Table S1. Problematic Use Composite item statistics (n = 364)**

Eight statements rated on a five-point Likert scale (1 = strongly disagree, 5 = strongly agree). Three items worded in the direction of controlled use were reverse-scored before summation. Scores were summed up to give a total from 8 to 40, with higher values indicating more problematic use.

| Item | Scoring | Mean (SD) | Corrected item–total r | α if item deleted |
| --- | --- | --- | --- | --- |
| I often use social media longer than I intended | Direct | 3.14 (1.37) | 0.669 | 0.762 |
| I feel anxious or restless when I cannot access social media | Direct | 3.20 (1.27) | 0.486 | 0.790 |
| I have missed sleep because of social media use | Direct | 3.08 (1.42) | 0.661 | 0.763 |
| I check social media as a way to cope with academic stress | Direct | 3.24 (1.27) | 0.469 | 0.793 |
| My sleep quality has worsened since I started using social media heavily | Direct | 3.08 (1.37) | 0.660 | 0.763 |
| Social media has helped me perform better academically | Reversed | 2.91 (1.31) | 0.333 | 0.811 |
| I am aware of how much time I spend on social media daily | Reversed | 2.96 (1.30) | 0.407 | 0.801 |
| I have set deliberate limits on my social media use | Reversed | 2.90 (1.33) | 0.476 | 0.792 |

*Scale statistics. Cronbach’s α = 0.807. Theoretical range 8–40; observed range 8–40. Mean 24.51 (SD 6.95). The Problematic Use Composite is a de novo composite constructed for this study and is not a validated instrument. It was analyzed as a secondary, exploratory measure, was not used to define groups, and no clinical threshold was applied. Reversed items are shown with their reverse-scored item statistics; reverse scoring was applied before summation.*

**Supplementary Figure S1. Conceptual framework diagram informing covariate selection.** The diagram was specified before modeling and retained as a conceptual framework for relationships among background characteristics, social-media behaviors and the outcomes. Background characteristics were included as covariates plausibly related to both social media behavior and the outcomes. The five social-media variables were treated as exposures of interest and are grouped here into the three exposure dimensions examined in this study: amount, purpose, and timing and context. Every exposure dimension is drawn with a path to both outcomes, because each dimension was tested against both outcomes in the same mutually adjusted models. The arrows denote relationships hypothesized before analysis; they do not depict estimated associations, and no path is annotated with a study result. Sleep quality is shown as potentially related to academic performance and was therefore not included as a covariate in the academic model. The dashed path depicts a hypothesized relationship between purpose and timing of use for conceptual purposes; no mediation analysis was performed, and the cross-sectional design does not establish temporal or causal pathways.

Depression, anxiety, caffeine use, call or shift duties, commuting time and pre-existing sleep disorders were not measured and are not encoded in the graph; each could confound any of the paths shown.

**Supplementary Note S1. Model specifications reported in this article.**

| Model specification | Adjusts for | Interpretation |
| --- | --- | --- |
| Minimally adjusted | Background covariates only | Adjusted association of each exposure without conditioning on the other social-media exposures |
| Mutually adjusted (primary) | Background covariates + all other measured social-media exposures | Adjusted association of each exposure conditional on the other measured social-media exposures |

**Supplementary Table S2. Platform-by-purpose cross-tabulation (n = 364)**

Percentages are calculated within each platform’s Q14-defined user base. Q14 and Q15 were consistency-checked so that each recorded primary-purpose response corresponded to a platform marked as used.

| Platform | Users, n | Academic / study | Communication with peers | Entertainment / leisure | News / information | Mixed — no clear primary |
| --- | --- | --- | --- | --- | --- | --- |
| YouTube | 356 | 161 (45.2) | 6 (1.7) | 155 (43.5) | 20 (5.6) | 14 (3.9) |
| WhatsApp | 353 | 135 (38.2) | 153 (43.3) | 18 (5.1) | 25 (7.1) | 22 (6.2) |
| Instagram | 302 | 19 (6.3) | 37 (12.3) | 206 (68.2) | 27 (8.9) | 13 (4.3) |
| TikTok | 224 | 14 (6.2) | 8 (3.6) | 173 (77.2) | 14 (6.2) | 15 (6.7) |
| Facebook | 198 | 18 (9.1) | 39 (19.7) | 74 (37.4) | 55 (27.8) | 12 (6.1) |
| Snapchat | 143 | 0 (0.0) | 6 (4.2) | 94 (65.7) | 2 (1.4) | 41 (28.7) |
| LinkedIn | 114 | 72 (63.2) | 16 (14.0) | 4 (3.5) | 17 (14.9) | 5 (4.4) |
| Twitter/X | 82 | 7 (8.5) | 6 (7.3) | 16 (19.5) | 47 (57.3) | 6 (7.3) |

*Values are n (%). Bold indicates the most frequently reported purpose for each platform.*

**Supplementary Table S3. Spearman correlation p-value matrix (n = 364)**

Coefficients are given in Table 4 of the main text. Variables: 1 total daily use; 2 academic time %; 3 study-time multitasking; 4 duration of pre-sleep social-media use; 5 nocturnal checking; 6 Problematic Use Composite; 7 number of platforms; 8 PSQI global score; 9 examination percentage.

| Pair | ρ | p |
| --- | --- | --- |
| 1 × 2 | −0.514 | 6.0e−26 |
| 1 × 3 | 0.519 | 1.6e−26 |
| 1 × 4 | 0.629 | 1.9e−41 |
| 1 × 5 | 0.306 | 2.4e−9 |
| 1 × 6 | 0.718 | 5.7e−59 |
| 1 × 7 | 0.324 | 2.3e−10 |
| 1 × 8 | 0.360 | 1.5e−12 |
| 1 × 9 | −0.441 | 1.0e−18 |
| 2 × 3 | −0.389 | 1.4e−14 |
| 2 × 4 | −0.417 | 9.5e−17 |
| 2 × 5 | −0.304 | 3.2e−9 |
| 2 × 6 | −0.636 | 1.3e−42 |
| 2 × 7 | −0.155 | 0.0029375 |
| 2 × 8 | −0.282 | 4.3e−8 |
| 2 × 9 | 0.506 | 5.1e−25 |
| 3 × 4 | 0.443 | 6.7e−19 |
| 3 × 5 | 0.174 | 0.00087065 |
| 3 × 6 | 0.553 | 1.7e−30 |
| 3 × 7 | 0.164 | 0.0016802 |
| 3 × 8 | 0.315 | 7.7e−10 |
| 3 × 9 | −0.319 | 4.7e−10 |
| 4 × 5 | 0.364 | 7.4e−13 |
| 4 × 6 | 0.691 | 6.2e−53 |
| 4 × 7 | 0.290 | 1.7e−8 |
| 4 × 8 | 0.485 | 7.0e−23 |
| 4 × 9 | −0.386 | 2.4e−14 |
| 5 × 6 | 0.380 | 5.9e−14 |
| 5 × 7 | 0.091 | 0.082987 |
| 5 × 8 | 0.203 | 9.8e−5 |
| 5 × 9 | −0.231 | 8.3e−6 |
| 6 × 7 | 0.243 | 2.8e−6 |
| 6 × 8 | 0.440 | 1.2e−18 |
| 6 × 9 | −0.530 | 9.4e−28 |
| 7 × 8 | 0.170 | 0.0011165 |
| 7 × 9 | −0.108 | 0.039891 |
| 8 × 9 | −0.166 | 0.0014725 |

*All tests two-sided. 35 of the 36 pairwise correlations were significant at p < 0.05; the exception was nocturnal checking × number of platforms (ρ = 0.091, p = 0.083).*

**Supplementary Table S4. Linear regression model for the PSQI global score (n = 364)**

| Predictor | B | SE | Std β | 95% CI | t | p |
| --- | --- | --- | --- | --- | --- | --- |
| Intercept | 2.311 | 0.962 | — | 0.418 to 4.203 | 2.402 | 0.017 |
| Male sex | 0.239 | 0.340 | 0.033 | −0.429 to 0.907 | 0.703 | 0.483 |
| 3rd year | 1.093 | 0.506 | 0.134 | 0.099 to 2.087 | 2.160 | 0.031 |
| 4th year | 0.809 | 0.510 | 0.099 | −0.193 to 1.812 | 1.586 | 0.113 |
| Final year | 0.597 | 0.513 | 0.073 | −0.411 to 1.606 | 1.164 | 0.245 |
| MBBS program | 0.174 | 0.364 | 0.022 | −0.542 to 0.889 | 0.478 | 0.634 |
| Private institution | 0.149 | 0.375 | 0.019 | −0.588 to 0.887 | 0.397 | 0.691 |
| Hostel / on-campus residence | 0.283 | 0.344 | 0.038 | −0.395 to 0.960 | 0.823 | 0.412 |
| Total daily social media use | 0.127 | 0.176 | 0.047 | −0.220 to 0.473 | 0.722 | 0.473 |
| Purpose: mixed | 0.456 | 0.524 | 0.049 | −0.575 to 1.487 | 0.870 | 0.385 |
| Purpose: entertainment-dominated | 0.514 | 0.427 | 0.070 | −0.325 to 1.353 | 1.204 | 0.229 |
| Study-time multitasking | 0.278 | 0.199 | 0.077 | −0.112 to 0.669 | 1.397 | 0.162 |
| Duration of pre-sleep social-media use | 1.594 | 0.261 | 0.392 | 1.081 to 2.106 | 6.107 | < 0.001 |
| Nocturnal checking | 0.108 | 0.209 | 0.026 | −0.303 to 0.519 | 0.517 | 0.607 |

*R² = 0.263, adjusted R² = 0.235, F(13, 350) = 9.58, p < 0.001. Reference categories: female, 2nd year, BDS, public institution, off-campus residence, academic purpose.*

**Supplementary Table S5. Linear regression model for examination percentage (n = 364)**

| Predictor | B | SE | Std β | 95% CI | t | p |
| --- | --- | --- | --- | --- | --- | --- |
| Intercept | 90.142 | 1.802 | — | 86.598 to 93.686 | 50.023 | < 0.001 |
| Male sex | 0.121 | 0.636 | 0.009 | −1.130 to 1.372 | 0.190 | 0.849 |
| 3rd year | −0.441 | 0.947 | −0.029 | −2.303 to 1.421 | −0.466 | 0.642 |
| 4th year | −0.722 | 0.955 | −0.047 | −2.600 to 1.156 | −0.756 | 0.450 |
| Final year | −0.118 | 0.960 | −0.008 | −2.007 to 1.771 | −0.123 | 0.902 |
| MBBS program | 1.565 | 0.682 | 0.107 | 0.225 to 2.906 | 2.295 | 0.022 |
| Private institution | −0.302 | 0.702 | −0.020 | −1.683 to 1.079 | −0.430 | 0.668 |
| Hostel / on-campus residence | −0.990 | 0.645 | −0.072 | −2.259 to 0.278 | −1.535 | 0.126 |
| Total daily social media use | −1.404 | 0.330 | −0.282 | −2.053 to −0.756 | −4.255 | < 0.001 |
| Purpose: mixed | −0.489 | 0.982 | −0.028 | −2.420 to 1.441 | −0.498 | 0.618 |
| Purpose: entertainment-dominated | −0.928 | 0.799 | −0.068 | −2.499 to 0.644 | −1.161 | 0.246 |
| Study-time multitasking | −0.541 | 0.372 | −0.080 | −1.272 to 0.190 | −1.454 | 0.147 |
| Duration of pre-sleep social-media use | −0.915 | 0.488 | −0.121 | −1.875 to 0.045 | −1.875 | 0.062 |
| Nocturnal checking | −0.628 | 0.392 | −0.082 | −1.398 to 0.142 | −1.602 | 0.110 |

R² = 0.254, adjusted R² = 0.226, F(13, 350) = 9.17, p < 0.001. Reference categories as in Supplementary Table S4.

**Supplementary Table S6. Program-stratified linear regression models**

Fitted separately in MBBS and BDS students using the same specification as the primary models, with program omitted. Behavioral exposures only are shown; demographic covariates (sex, year of study, institution type, residence) were included in every model.

### S6a. PSQI global score

| Predictor | MBBS (n = 252) B (95% CI), p | BDS (n = 112) B (95% CI), p |
| --- | --- | --- |
| Total daily social media use | 0.056 (−0.379 to 0.492), p = 0.799 | 0.385 (−0.241 to 1.011), p = 0.225 |
| Purpose: mixed | 0.459 (−0.809 to 1.727), p = 0.477 | 0.991 (−0.912 to 2.895), p = 0.304 |
| Purpose: entertainment | 0.418 (−0.598 to 1.433), p = 0.419 | 1.168 (−0.479 to 2.815), p = 0.162 |
| Study-time multitasking | 0.219 (−0.258 to 0.696), p = 0.367 | 0.267 (−0.469 to 1.003), p = 0.474 |
| Duration of pre-sleep social-media use | 1.658 (1.039 to 2.277), p = < 0.001 | 1.235 (0.226 to 2.244), p = 0.017 |
| Nocturnal checking | −0.039 (−0.524 to 0.447), p = 0.876 | 0.505 (−0.369 to 1.379), p = 0.254 |
| Model | R² = 0.263, F(12, 239) = 7.11, p < 0.001 | R² = 0.309, F(12, 99) = 3.7, p < 0.001 |

### S6b. Examination percentage

| Predictor | MBBS (n = 252) B (95% CI), p | BDS (n = 112) B (95% CI), p |
| --- | --- | --- |
| Total daily social media use | −1.303 (−2.115 to −0.490), p = 0.002 | −1.424 (−2.571 to −0.277), p = 0.015 |
| Purpose: mixed | 0.636 (−1.730 to 3.001), p = 0.597 | −2.611 (−6.099 to 0.877), p = 0.141 |
| Purpose: entertainment | −0.572 (−2.466 to 1.323), p = 0.553 | −1.415 (−4.433 to 1.602), p = 0.354 |
| Study-time multitasking | −0.486 (−1.376 to 0.403), p = 0.283 | −0.462 (−1.812 to 0.887), p = 0.498 |
| Duration of pre-sleep social-media use | −0.775 (−1.929 to 0.379), p = 0.187 | −1.655 (−3.504 to 0.194), p = 0.079 |
| Nocturnal checking | −1.268 (−2.174 to −0.362), p = 0.006 | 1.193 (−0.408 to 2.795), p = 0.142 |
| Model | R² = 0.273, F(12, 239) = 7.47, p < 0.001 | R² = 0.269, F(12, 99) = 3.04, p = 0.001 |

Formal interaction tests (full sample). Program × exposure interaction terms for all five behavioral exposures were non-significant for both outcomes: PSQI global score, incremental F(6, 344) = 0.457, p = 0.840, ΔR² = 0.006; examination percentage, F(6, 344) = 1.716, p = 0.116, ΔR² = 0.022. The six numerator degrees of freedom comprise one term for each of total daily use, study-time multitasking, duration of pre-sleep social-media use and nocturnal checking, and two for the three-category purpose variable.

*The direction of association was consistent across programs for duration of pre-sleep social-media use and for total daily use, the two principal findings: longer duration of pre-sleep social-media use was associated with poorer sleep in both strata, and total daily use with lower examination marks in both. Differences in statistical significance between strata reflect the reduced precision of the smaller BDS subgroup rather than evidence of effect modification, as indicated by the non-significant interaction tests. Stratified estimates are provided for transparency and should not be interpreted as evidence that associations differ by program.*

**Supplementary Table S7. Maximum-likelihood and Firth penalized-likelihood logistic regression for poor sleep quality (PSQI > 5) (n = 364)**

| Predictor | Maximum likelihood OR (95% CI) | p | Firth penalized likelihood OR (95% CI) | p |
| --- | --- | --- | --- | --- |
| Total daily social media use (per category) | 0.97 (0.68–1.37) | 0.844 | 0.97 (0.69–1.35) | 0.838 |
| Purpose: mixed (ref. academic) | 2.66 (0.97–7.30) | 0.057 | 2.44 (0.93–6.36) | 0.060 |
| Purpose: entertainment-dominated (ref. academic) | 2.58 (1.22–5.43) | 0.013 | 2.46 (1.19–5.06) | 0.015 |
| Study-time multitasking (per category) | 0.99 (0.63–1.53) | 0.949 | 1.00 (0.66–1.51) | 0.992 |
| Duration of pre-sleep social-media use (per category) | 2.70 (1.66–4.38) | < 0.001 | 2.53 (1.59–4.04) | < 0.001 |
| Nocturnal checking (per category) | 1.03 (0.72–1.48) | 0.873 | 1.03 (0.73–1.47) | 0.860 |
| Male sex (ref. female) | 1.15 (0.59–2.22) | 0.684 | 1.14 (0.61–2.16) | 0.682 |
| 3rd year (ref. 2nd year) | 2.55 (1.01–6.48) | 0.048 | 2.41 (0.98–5.91) | 0.053 |
| 4th year (ref. 2nd year) | 2.50 (0.94–6.65) | 0.067 | 2.35 (0.92–6.00) | 0.073 |
| Final year (ref. 2nd year) | 1.67 (0.69–4.07) | 0.255 | 1.62 (0.68–3.84) | 0.269 |
| MBBS program (ref. BDS) | 1.13 (0.56–2.29) | 0.724 | 1.14 (0.58–2.24) | 0.709 |
| Private institution (ref. public) | 0.58 (0.27–1.25) | 0.166 | 0.61 (0.29–1.28) | 0.182 |
| Hostel / on-campus residence (ref. off-campus) | 1.40 (0.70–2.80) | 0.342 | 1.36 (0.70–2.64) | 0.368 |

*OR, odds ratio; CI, confidence interval; PSQI, Pittsburgh Sleep Quality Index. Both models contain the same 13 predictor degrees of freedom. The outcome has 312 poor sleepers and 52 good sleepers, so the limiting outcome category yields 4.0 events per variable; Firth’s penalized likelihood was fitted as a sensitivity analysis because it is robust to sparse-data bias. Firth confidence intervals are Wald intervals from the penalized fit, with p-values from the corresponding Wald tests. No coefficient changed sign. One predictor crossed the α = 0.05 boundary: third year relative to second year (maximum likelihood OR 2.55, p = 0.048; Firth OR 2.41, p = 0.053).*

**Supplementary Table S8. Secondary linear regression models including academic-use proportion (Q16) (n = 364)**

Adds academic-use proportion (the proportion of daily social media time spent on academic or study-related purposes, questionnaire item Q16, coded 1–6 as in Table 4 of the main text) to the mutually adjusted primary models reported in full in Supplementary Tables S4 and S5, as described in Section 2.8. All other predictors and reference categories are as in those tables.

### S8a. PSQI global score

| Predictor | B | SE | Std β | 95% CI | t | p |
| --- | --- | --- | --- | --- | --- | --- |
| Intercept | 3.634 | 1.406 | — | 0.870 to 6.399 | 2.585 | 0.010 |
| Male sex | 0.255 | 0.339 | 0.035 | −0.413 to 0.922 | 0.752 | 0.454 |
| 3rd year | 1.149 | 0.507 | 0.141 | 0.152 to 2.146 | 2.266 | 0.024 |
| 4th year | 0.846 | 0.510 | 0.104 | −0.157 to 1.850 | 1.659 | 0.098 |
| Final year | 0.652 | 0.514 | 0.080 | −0.359 to 1.663 | 1.268 | 0.206 |
| MBBS program | 0.210 | 0.365 | 0.027 | −0.507 to 0.928 | 0.575 | 0.565 |
| Private institution | 0.117 | 0.375 | 0.015 | −0.622 to 0.855 | 0.312 | 0.756 |
| Hostel / on-campus residence | 0.233 | 0.346 | 0.031 | −0.448 to 0.914 | 0.673 | 0.502 |
| Total daily social media use | 0.061 | 0.183 | 0.023 | −0.299 to 0.421 | 0.333 | 0.738 |
| Purpose: mixed | 0.402 | 0.525 | 0.043 | −0.631 to 1.436 | 0.766 | 0.444 |
| Purpose: entertainment-dominated | 0.392 | 0.437 | 0.053 | −0.466 to 1.251 | 0.897 | 0.370 |
| Study-time multitasking | 0.240 | 0.201 | 0.066 | −0.155 to 0.634 | 1.194 | 0.233 |
| Duration of pre-sleep social-media use | 1.576 | 0.261 | 0.387 | 1.063 to 2.089 | 6.038 | < 0.001 |
| Nocturnal checking | 0.066 | 0.211 | 0.016 | −0.350 to 0.482 | 0.313 | 0.755 |
| Academic-use proportion (Q16) | −0.236 | 0.182 | −0.075 | −0.594 to 0.123 | −1.297 | 0.198 |

R² = 0.266, adjusted R² = 0.237, F(14, 349) = 9.04, p < 0.001. Reference categories as in Supplementary Table S4.

### S8b. Examination percentage

| Predictor | B | SE | Std β | 95% CI | t | p |
| --- | --- | --- | --- | --- | --- | --- |
| Intercept | 78.071 | 2.485 | — | 73.183 to 82.959 | 31.417 | < 0.001 |
| Male sex | −0.026 | 0.600 | −0.002 | −1.206 to 1.155 | −0.043 | 0.966 |
| 3rd year | −0.951 | 0.896 | −0.063 | −2.714 to 0.812 | −1.061 | 0.289 |
| 4th year | −1.058 | 0.902 | −0.070 | −2.832 to 0.717 | −1.173 | 0.242 |
| Final year | −0.616 | 0.909 | −0.041 | −2.404 to 1.172 | −0.678 | 0.498 |
| MBBS program | 1.231 | 0.645 | 0.084 | −0.037 to 2.500 | 1.909 | 0.057 |
| Private institution | −0.006 | 0.664 | −0.000 | −1.312 to 1.299 | −0.009 | 0.993 |
| Hostel / on-campus residence | −0.535 | 0.612 | −0.039 | −1.740 to 0.669 | −0.874 | 0.383 |
| Total daily social media use | −0.809 | 0.324 | −0.162 | −1.445 to −0.172 | −2.497 | 0.013 |
| Purpose: mixed | −0.000 | 0.929 | −0.000 | −1.827 to 1.827 | −0.000 | 1.000 |
| Purpose: entertainment-dominated | 0.184 | 0.772 | 0.013 | −1.334 to 1.703 | 0.238 | 0.812 |
| Study-time multitasking | −0.187 | 0.355 | −0.028 | −0.885 to 0.511 | −0.527 | 0.598 |
| Duration of pre-sleep social-media use | −0.754 | 0.461 | −0.100 | −1.660 to 0.153 | −1.636 | 0.103 |
| Nocturnal checking | −0.247 | 0.374 | −0.032 | −0.982 to 0.489 | −0.660 | 0.510 |
| Academic-use proportion (Q16) | 2.148 | 0.323 | 0.369 | 1.513 to 2.782 | 6.650 | < 0.001 |

R² = 0.338, adjusted R² = 0.311, F(14, 349) = 12.73, p < 0.001. Reference categories as in Supplementary Table S4.

These two models add academic-use proportion to the primary mutually adjusted models in Supplementary Tables S4 and S5. All variance inflation factors remained below 2.23 and maximum leverage was 0.076, indicating that adding this variable introduced no meaningful collinearity. The academic-use-proportion and total-daily-use coefficients from the examination-percentage model, and the academic-use-proportion coefficient from the PSQI model, are summarized in Section 3.7 of the main text. One further observation follows from the full table here: the MBBS-program coefficient, significant in the primary examination-percentage model (Supplementary Table S5, p = 0.022), is attenuated to borderline significance once academic-use proportion is added (B = 1.231, p = 0.057).

**Supplementary Table S9. Sensitivity analyses treating ordinal exposures as categorical factors**

| **Exposure / comparison** | **PSQI global score** | **Examination percentage** |
| --- | --- | --- |
| Total daily social media use | F(5, 340) = 0.75, p = 0.590 | F(5, 340) = 3.49, p = 0.004 |
| Study-time multitasking | F(3, 340) = 2.81, p = 0.039 | F(3, 340) = 1.24, p = 0.296 |
| Duration of pre-sleep social-media use | F(3, 340) = 11.64, p < 0.001 | F(3, 340) = 1.34, p = 0.262 |
| Nocturnal checking | F(3, 340) = 0.57, p = 0.633 | F(3, 340) = 1.01, p = 0.388 |
| Categorical vs linear-per-category specification | F(10, 340) = 1.46, p = 0.154 | F(10, 340) = 0.48, p = 0.901 |

The four primary ordinal exposures were entered simultaneously as categorical factors using indicator variables, with the lowest observed category as the reference; the F tests above assess each factor jointly. The categorical models had R² = 0.293 for the PSQI global score and R² = 0.264 for examination percentage. The final row compares each categorical model with its corresponding linear-per-category primary model. In a separate secondary sensitivity model, academic-use proportion (Q16) was entered as a categorical factor while all other terms retained their primary coding: Q16 remained associated with examination percentage (F(5, 345) = 9.63, p < 0.001) but not with the PSQI global score (F(5, 345) = 0.65, p = 0.659), and total daily use remained associated with examination percentage (B = −0.828, p = 0.011).

**Supplementary Table S10. Outcome specificity: difference between the sleep and academic associations (n = 364; 728 stacked records)**

Each participant contributes two records, one per outcome. The PSQI global score and the reversed examination percentage were each standardized to mean 0 and SD 1, so that a positive coefficient denotes a worse outcome on both, and the four ordinal exposures were standardized, so coefficients are standardized betas. A single model regresses the stacked outcome on outcome type interacted with every term of the primary specification (Supplementary Tables S4 and S5), with standard errors clustered on participant. The difference column is the outcome × exposure interaction: the standardized association with the reversed examination outcome minus the standardized association with sleep quality. Method described in Section 2.8; results in Section 3.12.

| **Exposure** | **β, sleep quality** | **β, examination performance** | **Difference (95% CI)** | **p** | **Holm-adjusted p** |
| --- | --- | --- | --- | --- | --- |
| Duration of pre-sleep social-media use | 0.392 | 0.121 | −0.271 (−0.463 to −0.079) | 0.006 | 0.023 |
| Total daily social media use | 0.047 | 0.282 | 0.235 (0.032 to 0.437) | 0.023 | 0.069 |
| Study-time multitasking | 0.077 | 0.080 | 0.003 (−0.149 to 0.156) | 0.966 | 0.966 |
| Nocturnal checking | 0.026 | 0.082 | 0.056 (−0.083 to 0.195) | 0.429 | 0.857 |
| Purpose: mixed vs academic | 0.125 | 0.072 | −0.053 (−0.454 to 0.348) | 0.795 | — |
| Purpose: entertainment-dominated vs academic | 0.141 | 0.137 | −0.004 (−0.355 to 0.346) | 0.980 | — |

Joint Wald test of the two exposures carrying the principal findings (duration of pre-sleep use and total daily use): χ²(2) = 8.30, p = 0.016. Joint Wald test of all four behavioral exposures: χ²(4) = 8.59, p = 0.072; the larger test includes two exposures for which no difference between outcomes was expected. Holm–Bonferroni adjustment is across the four behavioral exposures. Under that adjustment the pre-sleep contrast remains significant and the total-use contrast is attenuated to p = 0.069, so the evidence for the total-use contrast is the weaker of the two. Because the model is saturated in the outcome dimension, the point estimates in the first two columns are identical to those obtained from the two separate primary models; what this specification adds is a test of the difference between them.

**Supplementary Table S11. Criterion-overlap sensitivity analyses for duration of pre-sleep social-media use (n = 364)**

Duration of pre-sleep social-media use and three PSQI components — sleep latency (component 2), sleep duration (component 3) and habitual sleep efficiency (component 4) — refer to the same interval of the night, so an association between them could in principle reflect definitional overlap between exposure and outcome rather than a substantive relationship. Panel A refits the primary mutually adjusted sleep model (Supplementary Table S4) on restricted versions of the outcome; panel B models each component separately. All models contain the same 13 predictor degrees of freedom and the same reference categories as Supplementary Table S4; only the coefficient for duration of pre-sleep social-media use is shown.

### S11a. Restricted sleep outcomes

| **Outcome** | **Range** | **Mean (SD)** | **B (95% CI)** | **p** | **Standardized β** | **Model R²** |
| --- | --- | --- | --- | --- | --- | --- |
| PSQI global score (all seven components) | 0–21 | 10.09 (3.64) | 1.594 (1.081 to 2.106) | < 0.001 | 0.392 | 0.263 |
| PSQI excluding component 2 (sleep latency) | 0–18 | 8.63 (3.26) | 1.398 (0.936 to 1.859) | < 0.001 | 0.384 | 0.255 |
| PSQI excluding components 2, 3 and 4 | 0–12 | 5.28 (1.95) | 0.767 (0.487 to 1.047) | < 0.001 | 0.352 | 0.234 |
| Components 5, 6 and 7 only | 0–9 | 3.72 (1.33) | 0.382 (0.183 to 0.580) | < 0.001 | 0.257 | 0.169 |

### S11b. Individual PSQI components

| **PSQI component** | **B (95% CI)** | **p** |
| --- | --- | --- |
| 1 Subjective sleep quality | 0.386 (0.221 to 0.550) | < 0.001 |
| 2 Sleep latency | 0.196 (0.093 to 0.298) | < 0.001 |
| 3 Sleep duration | 0.293 (0.181 to 0.405) | < 0.001 |
| 4 Habitual sleep efficiency | 0.338 (0.199 to 0.477) | < 0.001 |
| 5 Sleep disturbance | 0.060 (−0.023 to 0.142) | 0.156 |
| 6 Sleep medication use | 0.021 (−0.059 to 0.100) | 0.611 |
| 7 Daytime dysfunction | 0.301 (0.169 to 0.434) | < 0.001 |

The association is preserved when every component referring to time in bed is removed from the outcome, the standardized coefficient falling only from 0.392 to 0.257. The exposure was associated with five of the seven components but not individually with sleep disturbance (p = 0.156) or medication use (p = 0.611). Definitional overlap therefore does not account for the finding reported in Sections 3.6 and 4.2.

**Supplementary Table S12. Clustering sensitivity analysis: reported estimates with standard errors inflated by the design effect (n = 364)**

Participants were recruited from ten institutions, but the institution of enrollment was not recorded at participant level, so the intracluster correlation (ρ) could not be estimated and cluster-robust standard errors could not be fitted (Section 2.3). This table therefore reports an arithmetic sensitivity analysis rather than a refitted multilevel model. The standard error implied by each reported confidence interval was multiplied by √DEFF, where DEFF = 1 + (m̄ − 1)ρ and m̄ = 36.4 participants per institution, and the interval was recomputed. The two values of ρ are assumed, not estimated: at ρ = 0.01, DEFF = 1.354 and standard errors are 16.4% larger; at ρ = 0.02, DEFF = 1.708 and standard errors are 30.7% larger, corresponding to effective sample sizes of 269 and 213. Logistic estimates were inflated on the log-odds scale and back-transformed. The exercise gives a lower bound on the true uncertainty, not an estimate of it (Section 4.9). Results are summarized in Section 3.11.

| **Estimate** | **Reported 95% CI** | **Widened 95% CI, ρ = 0.01** | **Widened 95% CI, ρ = 0.02** | **Excludes the null** |
| --- | --- | --- | --- | --- |
| Pre-sleep use → PSQI global score (B = 1.594) | 1.081 to 2.106 | 0.998 to 2.190 | 0.924 to 2.264 | Both |
| Total daily use → examination percentage (B = −1.404) | −2.053 to −0.756 | −2.159 to −0.649 | −2.252 to −0.556 | Both |
| Academic-use proportion → examination percentage, secondary model (B = 2.148) | 1.513 to 2.782 | 1.410 to 2.886 | 1.319 to 2.977 | Both |
| Pre-sleep use → poor sleep quality, logistic model (aOR 2.70) | 1.66 to 4.38 | 1.54 to 4.75 | 1.43 to 5.09 | Both |
| Outcome-specificity contrast, pre-sleep use (Δβ = −0.271) | −0.463 to −0.079 | −0.494 to −0.048 | −0.522 to −0.020 | Both |
| MBBS vs BDS → examination percentage (B = 1.565) | 0.225 to 2.906 | 0.006 to 3.126 | −0.186 to 3.318 | ρ = 0.01 only |
| Total daily use → examination percentage, secondary model (B = −0.809) | −1.445 to −0.172 | −1.550 to −0.068 | −1.641 to 0.023 | ρ = 0.01 only |
| Entertainment-dominated purpose → poor sleep quality, logistic model (aOR 2.58) | 1.22 to 5.43 | 1.08 to 6.15 | 0.97 to 6.84 | ρ = 0.01 only |
| Third year vs second year → PSQI global score (B = 1.093) | 0.099 to 2.087 | −0.064 to 2.250 | −0.206 to 2.392 | Neither |
| Outcome-specificity contrast, total daily use (Δβ = 0.235) | 0.032 to 0.437 | −0.001 to 0.471 | −0.030 to 0.500 | Neither |

**Supplementary Table S13. Questionnaire items, response options and coding for every variable analyzed (n = 364)**

This table sets out, for every variable analyzed in the article, the response options offered and the coding applied, relocated from Section 2.5 of the main text. Quoted text is the item stem as administered; where no stem is quoted, the main text described the construct and its options rather than reproducing the wording. Categories that no participant selected are marked, since they determine the observed range of the analyzed variable.

| **Variable** | **Response options as administered** | **Coding used in analysis** |
| --- | --- | --- |
| Age | Completed years, free text | Continuous (descriptive only) |
| Sex | Male / female / prefer not to say | Dichotomous; no participant selected “prefer not to say” |
| Year of study | Second (pre-clinical) / third / fourth / final (clinical) | Categorical, second year as reference |
| Program | MBBS / BDS | Binary, BDS as reference |
| Institution type | Public / private | Binary, public as reference |
| Living arrangement | Hostel or on-campus / off-campus or with family | Binary, off-campus as reference |
| Examination percentage | Free-text value on a 0–100 scale; instruction not to round to a band | Continuous outcome |
| Total daily social media use | < 30 min / 30 min–1 h / 1–2 h / 2–3 h / 3–5 h / > 5 h | Ordered, coded 1–6 |
| Years of social media use | Four ordered bands | Descriptive only |
| Platform use | Multiple-response over eight named platforms plus a free-text option | Count of platforms used, 0–8 |
| Primary purpose, per platform | Academic/study / communication with peers / entertainment/leisure / news/information / mixed — no clear primary | Input to the derived purpose variable below. Platforms not used were coded not applicable, and each response was consistency-checked against the platform-use item |
| Purpose of use (derived) | Not asked directly | Three categories: academic (more academic than entertainment nominations), entertainment-dominated (the converse) and mixed (equal non-zero counts). No participant had zero of both. Internal dataset labels: “Academic Purpose”, “Time Pass” and “Both” respectively |
| Academic-use proportion (Q16) | 0% / 1–20% / 21–40% / 41–60% / 61–80% / > 80% of total social media time | Ordered, coded 1–6; reported as the academic time percentage |
| Study-time multitasking | “How often do you use social media WHILE studying (multitasking)?” — five options from never (phone is away when I study) to always (continuously use social media while studying) | Ordered; “never” was selected by no participant, so the analyzed variable ranged 1–4 |
| Duration of pre-sleep social media use | “How much time do you spend on social media before sleeping?” — I do not use social media in the last hour before sleep / < 15 min / 15–30 min / 30–60 min / > 60 min | Ordered; the first option was selected by no participant, so the analyzed variable ranged 1–4 |
| Nocturnal checking | Frequency of checking social media on waking during the night: never / rarely / sometimes / often | Ordered, coded 1–4 |
| Phone location during sleep | Four categories | Descriptive only; “in another room” was selected by no participant |
| Sleep quality | Pittsburgh Sleep Quality Index, seven components (Section 2.6) | Global score 0–21 (primary outcome); > 5 dichotomy (secondary) |
| Problematic Use Composite | Eight statements, 1 = strongly disagree to 5 = strongly agree (Supplementary Table S1) | Three items reverse-scored, summed 8–40; exploratory only |

# Supplementary Note S2. Sample size, analytic adequacy and data handling

This note gives the calculations and supporting detail behind Sections 2.3 and 2.4 of the main text. It does not repeat the main text: each entry below reports only what is not stated there.

Precision achieved. The achieved half-width (Section 2.3) is narrower than the 5 percentage points planned because the observed proportion lies well away from 0.50; had the prevalence been 0.50, the half-width would have been 5.0 points without the finite population correction and 5.1 with it. The interval reported in Section 3.3 is a Wilson score interval without the correction, which is asymmetric about the point estimate and is the more conservative choice at proportions away from 0.50.

**Adequacy for multivariable models.** The 13 predictor degrees of freedom in each fully adjusted model are: total daily use; purpose of use (2 df); study-time multitasking; duration of pre-sleep social-media use; nocturnal checking; sex; year of study (3 df); program; institution type; and residence. The minimum detectable effects quoted in Section 2.3 correspond to R² = 0.048 for the omnibus F-test and to partial R² = 0.021 (semi-partial r = 0.15) for an individual predictor, so the study was sized to detect small effects by conventional benchmarks. For the group comparisons on examination percentage (SD 6.78), the smallest detectable difference at 80% power was 2.4 percentage points between the academic (n = 86) and entertainment-dominated (n = 210) groups, 2.6 points between the mixed (n = 68) and entertainment-dominated groups, and 3.1 points between the academic and mixed groups; the corresponding values for the PSQI global score (SD 3.64) were 1.3, 1.4 and 1.7 points.

**Adequacy for the logistic model.** Firth's penalized-likelihood refit (Section 2.3) left the estimates essentially unchanged: duration of pre-sleep social-media use OR 2.70 → 2.53 (95% CI 1.59 to 4.04) and entertainment-dominated purpose OR 2.58 → 2.46 (95% CI 1.19 to 5.06), with no coefficient changing sign. One predictor crossed the α = 0.05 boundary: third year relative to second year was marginally significant under maximum likelihood (OR 2.55, p = 0.048) and marginally non-significant under the penalized likelihood (OR 2.41, p = 0.053), a difference of no substantive consequence. Given the low events-per-variable ratio the penalized estimates are the more trustworthy of the two sets, and the binary model is interpreted throughout as secondary to the continuous one (Supplementary Table S7).

**Clustering.** Cochran's formula assumes simple random sampling, whereas participants were clustered within ten institutions. Applying the design effect given in Section 2.3 to the prevalence estimate widens its 95% confidence interval from ±3.6 to ±4.2 percentage points at an intracluster correlation of 0.01 and to ±4.7 points at 0.02. The only institution-level characteristic recorded at participant level was whether the institution was public or private, which is why no intracluster correlation could be estimated (Section 4.9); the consequences for the regression estimates are quantified in Supplementary Table S12.

# Supplementary Data Sheet 1. STROBE checklist for cross-sectional studies

Completed in accordance with von Elm E, Altman DG, Egger M, Pocock SJ, Gøtzsche PC, Vandenbroucke JP; STROBE Initiative. Lancet 2007;370:1453–7.

| Item | Recommendation | Reported in |
| --- | --- | --- |
| Title and abstract |  |  |
| 1a | Indicate the study’s design with a commonly used term in the title or the abstract | Title (“A Cross-Sectional Study”); Abstract, Methods |
| 1b | Provide in the abstract an informative and balanced summary of what was done and what was found | Abstract |
| Introduction |  |  |
| 2 | Explain the scientific background and rationale for the investigation being reported | Section 1, paragraphs 1–4 |
| 3 | State specific objectives, including any prespecified hypotheses | Section 1, final paragraph |
| Methods |  |  |
| 4 | Present key elements of study design early in the paper | Section 2.1 |
| 5 | Describe the setting, locations, and relevant dates, including periods of recruitment, exposure, follow-up, and data collection | Section 2.1 (ten named institutions, Khyber Pakhtunkhwa, October 2023 – April 2024) |
| 6a | Give the eligibility criteria, and the sources and methods of selection of participants | Sections 2.2 and 2.4 |
| 7 | Clearly define all outcomes, exposures, predictors, potential confounders, and effect modifiers. Give diagnostic criteria, if applicable | Sections 2.5.1–2.5.3 (exposures), 2.6 (outcomes), 2.8 and Supplementary Figure S1 (confounders) |
| 8 | For each variable of interest, give sources of data and details of methods of assessment. Describe comparability of assessment methods if there is more than one group | Sections 2.5 and 2.6 (all analyzed items, their wording and their response categories are defined in the main text) |
| 9 | Describe any efforts to address potential sources of bias | Sections 2.4 (recruitment script, anonymity of return, data entry and verification) and 2.8; Section 4.9 (selection bias, common-method variance, self-report) |
| 10 | Explain how the study size was arrived at | Section 2.3 (Cochran’s formula with finite population correction for the prevalence objective; adequacy for the multivariable models assessed separately via subjects-per-predictor, minimum detectable effect and events per variable; design effect for clustering considered) |
| 11 | Explain how quantitative variables were handled in the analyses. If applicable, describe which groupings were chosen and why | Sections 2.5.2 and 2.8 |
| 12a | Describe all statistical methods, including those used to control for confounding | Section 2.8 |
| 12b | Describe any methods used to examine subgroups and interactions | Section 2.8; program × exposure interaction tests, Section 3.11 and Supplementary Table S6 |
| 12c | Explain how missing data were addressed | Sections 2.4 and 3.1 (no missing values in the analytic dataset; complete-case analysis) |
| 12d | If applicable, describe analytical methods taking account of sampling strategy | Sections 2.3, 2.4 and 2.8; convenience sampling acknowledged and no weighting applied. Institution of enrollment was not recorded at participant level, so clustering was addressed by a design-effect sensitivity analysis (Section 3.11 and Supplementary Table S12) rather than by cluster-robust or multilevel estimation |
| 12e | Describe any sensitivity analyses | Section 2.8 and Section 3.11 (median regression, bootstrap confidence intervals, categorical coding of the ordinal exposures, criterion-overlap refits, and the clustering design-effect analysis reported in Supplementary Table S12) |
| Results |  |  |
| 13a | Report numbers of individuals at each stage of study — numbers potentially eligible, examined for eligibility, confirmed eligible, included in the study, completing follow-up, and analysed | Section 2.4 (391 approached → 12 declined → 379 returned → 15 incomplete → 364 analyzed) |
| 13b | Give reasons for non-participation at each stage | Section 2.4 (declined consent; incomplete core sections) |
| 13c | Consider use of a flow diagram | Participant flow reported numerically in Section 2.4; per-site figures not retained (stated in Sections 2.4 and 4.9) |
| 14a | Give characteristics of study participants and information on exposures and potential confounders | Table 1 |
| 14b | Indicate number of participants with missing data for each variable of interest | Section 2.4 (none) |
| 15 | Report numbers of outcome events or summary measures | Section 3.3 and Table 1 (312 poor sleepers, 85.7%); Table 1 (examination percentage) |
| 16a | Give unadjusted estimates and, if applicable, confounder-adjusted estimates and their precision. Make clear which confounders were adjusted for and why they were included | Tables 3–5 and Figure 1; Supplementary Tables S4–S5; Section 3.9 (minimally vs mutually adjusted associations); Section 2.8 and Supplementary Figure S1 for rationale |
| 16b | Report category boundaries when continuous variables were categorized | Sections 2.5.2 and 2.6 |
| 16c | If relevant, consider translating estimates of relative risk into absolute risk for a meaningful time period | Not applicable (cross-sectional; no time-to-event outcome) |
| 17 | Report other analyses done — e.g. analyses of subgroups and interactions, and sensitivity analyses | Sections 3.9–3.12; Supplementary Tables S6, S9, S11 and S12 (the last reporting the clustering sensitivity analysis) |
| Discussion |  |  |
| 18 | Summarise key results with reference to study objectives | Section 4.1 |
| 19 | Discuss limitations of the study, taking into account sources of potential bias or imprecision. Discuss both direction and magnitude of any potential bias | Section 4.9 (limitations, each with direction of bias where estimable, including clustering and instrument validation) |
| 20 | Give a cautious overall interpretation of results considering objectives, limitations, multiplicity of analyses, results from similar studies, and other relevant evidence | Sections 4.2–4.8 and Section 5 |
| 21 | Discuss the generalizability (external validity) of the study results | Section 4.9, “Generalisability”; Section 5 |
| Other information |  |  |
| 22 | Give the source of funding and the role of the funders for the present study and, if applicable, for the original study on which the present article is based | Funding statement |
